# Coarse-Grained Martini 3 Model of Chondroitin Sulfate A

**DOI:** 10.1021/acs.jctc.5c01743

**Published:** 2026-02-23

**Authors:** Paulius Greicius, Frauke Gräter, Fabian Grünewald, Camilo Aponte-Santamaría

**Affiliations:** † Max Planck Institute for Polymer Research, Ackermannweg 10, Mainz 55128, Germany; ‡ Max Planck School Matter to Life, Heidelberg 69120, Germany; § 40092 Heidelberg Institute for Theoretical Studies (HITS), Schloss-Wolfsbrunnenweg 35, Heidelberg 69118, Germany

## Abstract

Chondroitin sulfate A (CSA) is a negatively charged linear glycosaminoglycan that plays a vital role in many biological processes. Research on CSA has been challenging due to its size, chemical heterogeneity, and multitude of binding partners. To address these issues, we developed a model of CSA for coarse-grained molecular dynamics simulations based on the Martini 3 force field. We demonstrate that this model is capable of reproducing atomistic properties of the repeating CSA disaccharide unit, including its molecular volume, bonded interactions, and structural polymer properties of CSA chains of different lengths. In particular, for biologically relevant long chains and despite using an explicit solvent, the computational cost is significantly reduced, relative to the cost equivalent atomistic simulations would require. The compatibility of the model with the Martini Go̅ protein model was tested by retrieving the force–response relationship of the CSA–malaria adhesin VAR2CSA complex. Importantly, we explored the influence of electrostatics on CSA aggregation. We show that the default Martini 3 parameters lead to overaggregation. We provide at least three different strategies to alleviate this issue, making use of a bigger bead for sodium cations, reflecting their hydration shell, partial ionic charges as a mean-field resource to take into account electronic polarizability, and, optionally, particle mesh Ewald summation as a more robust treatment of long-range electrostatics. Our model enables predictive modeling of CSA and potentially other chondroitin sulfates with the Martini 3 force field. In addition, this model provides insights for the further development of coarse-grained models of highly charged systems.

## Graphical abstract

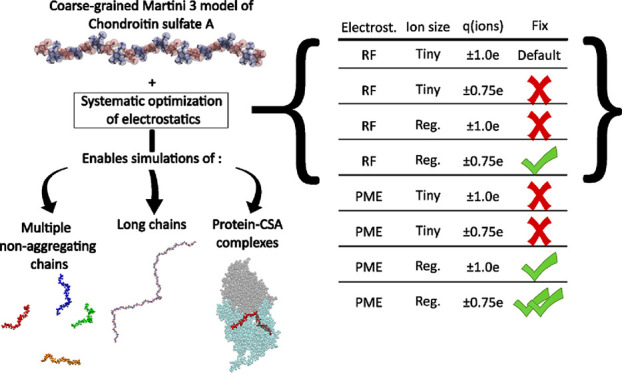

## Introduction

Chondroitin sulfate (CS) is a linear glycosaminoglycan composed of alternating *N*-acetyl galactosamine (GAL) and glucuronic acid (GLA) units connected via β1,3 and β1,4 linkages, respectively (Figure 1A). Sulfates can be variably added at various hydroxyls of the disaccharide, creating a highly negatively charged sugar. The polysaccharide is synthesized in the endoplasmic reticulum and Golgi, where it is also attached to cell surface or secreted proteins via serine residues.^1^ CS is a key extracellular matrix component in various tissues including skin,^2^ bone,^3^ cartilage,^4^ vasculature,^5^ cornea,^6^ and the central nervous system.^7^ Chains isolated from in vivo sources display high heterogeneity both in their molecular weight (50–150 kDa) and frequency of sulfated residues.^8^ Such structural diversity reflects the fact that CS serves a broad range of biological functions.

**Figure 1:**
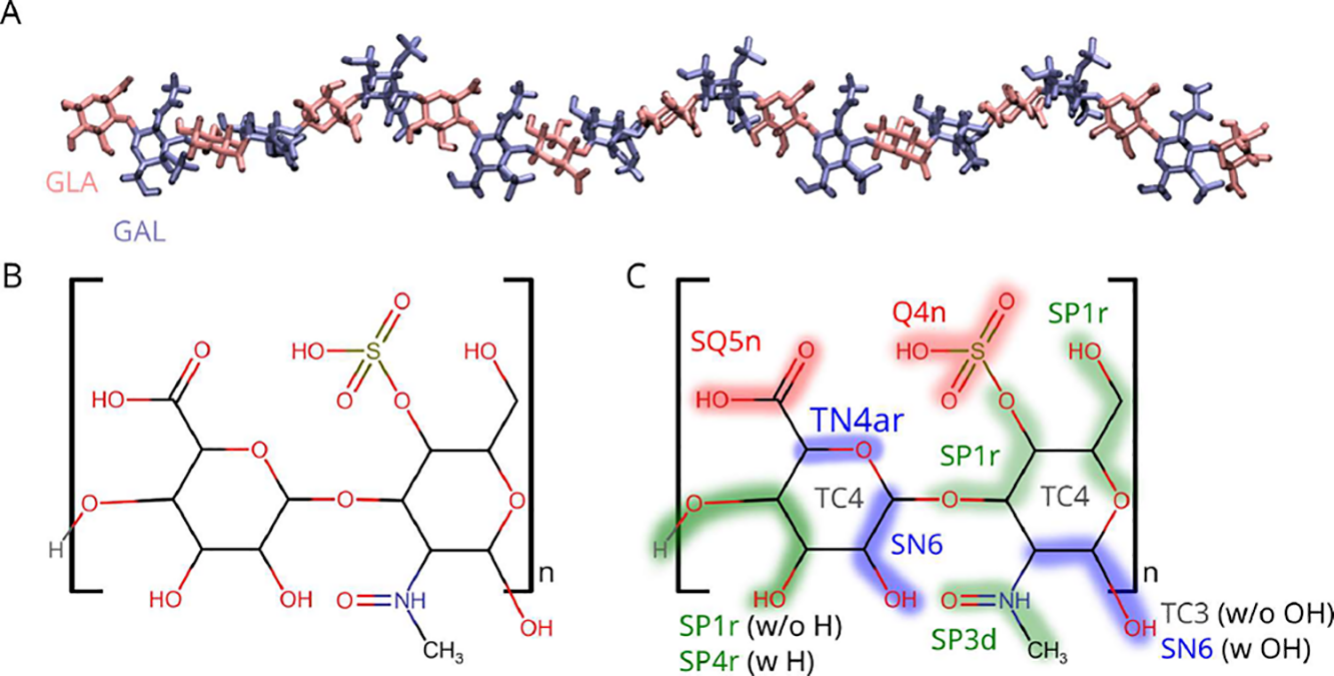
Structure and mapping of CSA into Martini 3 beads. (A) Structure of a CSA 21mer. d-Glucuronic acid (GLA) is colored in pink and *N*-acetyl-d-galactosamine 4-sulfate (GAL) in blue. (B) Chemical structure of the GLA–GAL repeating unit. (C) Mapping of the CSA repeating unit to Martini 3 beads. Shaded areas indicate atoms belonging to the same bead. Text next to each bead indicates the bead type. TC4 is the virtual site located in the center of the ring. Bead types that are part of the glycosidic bond are labeled “w/o”; meanwhile, beads at the end of the chain are labeled “w”.

The polyanionic nature and variable sulfation status of chondroitin sulfate chains enable them to form interactions with various cations^9^ or proteins.^1,7^ Heavily sulfated CS chains create high osmotic pressure, essential for load bearing in articulate cartilage,^10^ sustaining bone toughness,^11^ and hydration of cornea.^12^ Free carboxylate and sulfate groups chelate metal ions, especially divalent cations such as calcium.^13^ The ability of CS to sequester metal ions enhances its radical scavenging activity^14^ making it an effective antioxidant.^15^ Similarly, chelation of extracellular Ca^2+^ affects the transmembrane potential, enabling modulation of voltage-gated ion channel activity.^16^ The sulfation pattern on CS chains also dictates interactions with specific growth factors, morphogens, cytokines, or chemokines, thereby regulating various signaling pathways.^1,17,18^ In the nervous system, CS plays a role in axon growth and guidance, but depending on CS sulfation, it can either promote or inhibit these processes.^7,19^ Overall, although chondroitin sulfate has been implicated in many biological phenomena, it remains challenging to delineate the specific roles and properties of different CS chain types.

Chondroitin sulfate A (CSA), characterized by sulfate at carbon 4 of GAL residues (Figure 1B), is one of the most common CS types in animal tissues.^1^ It is the major type of CS in the human brain^20^ or at sites of spinal cord injury.^21^ CSA reduces with age in cartilage^4^ and has been associated with bone formation by facilitating collagen mineralization.^22^ The sugar is also involved in diseases: as a ligand for placental malaria pathogen^23,24^ and as a marker of cancer cells.^25^ In addition, CSA has received attention as a dietary supplement for osteoarthritis^26^ and as a component of hydrogels or drug delivery systems.^27^ However, research on this sugar is complicated by the heterogeneous nature of the samples,^8,28^ sometimes even leading to contradictory results.^19^ For this reason, an approach capable of studying well-defined, homogeneous CSA chains is needed to gain accurate insights into the sugar’s properties and interactions.

Molecular dynamics (MD) simulations have tremendously advanced our understanding of glycosaminoglycans, providing key aspects related to their structure, (thermo)­dynamics, energetics, and interactions (for comprehensive reviews, see Nagarajan et al.^29^ and Perez et al.^30^). The technique has the advantage of providing the spatial and temporal evolution of glycan systems at varying levels of resolution and with explicitly defined chain chemical composition. In the context of CS, MD has been applied to probe conformations of CS fragments with different sulfation motifs^31,32^ and the conformational impact of cation or hydroxyl radical binding.^33–35^ MD and molecular docking have also been employed to examine CS in complex with the chemokines CXCL8,^36^ CXCL14,^37^ the malarial protein VAR2CSA,^38–40^ and cysteine cathepsins.^41,42^ However, all of these studies employed CS chains of up to 20 monomer units of length due to the significant computational demands of all-atom MD simulations. In contrast, the chondroitin sulfate length in biological systems commonly exceeds 100 monomers.

One way to enable simulations of longer CS chains is by using coarse-grained (CG) MD simulations. In coarse-grained approximations, groups of atoms are represented as a single particle (pseudoatom), which reduces the number of entities within the system, thus speeding up its dynamics by reducing the total degrees of freedom. CG MD simulations of infinitely diluted chondroitin sulfate, represented by its backbone, have been shown to successfully reproduce experimental measurements including radius of gyration,^43^ osmotic pressure,^44^ and hydrodynamic radius.^45^ A CG model that also accounts for functional groups was introduced,^46^ but significant computational speedup was achieved only in implicit solvent. More recently, a CG model for the most common glycosaminoglycans, including fully sulfated CSs, has been developed in the Martini force field (version 2.2.).^47^ Nevertheless, a model for the most common CS form, i.e., CSA, with single sulfation at the fourth carbon of GAL, within the framework of generalized coarse-grained force fields for large-scale multicomponent simulations, is still needed.

Martini 3 is a widely used force field for coarse-grained simulations of biomolecules, with new and improved features over its predecessor Martini 2.2.^48^ Models for many carbohydrates have been implemented in the force field,^49–51^ but chondroitin sulfate has not yet been defined. In this study, we introduce a Martini 3 model for the CSA repeating unit which accurately reproduces the behavior of atomistic CHARMM36m^52^ simulations and examine the impact of simulation parameters on CSA aggregation. Then, we proceed to simulate CSA in complex with the CSA-binding protein VAR2CSA. Lastly, to illustrate the computational gains of the coarse-grained model, we perform equilibrium simulations of a 123-monomer-long CSA chain.

## Methods

### All-Atom Chondroitin Sulfate A Structure

A linear 21-subunit chondroitin sulfate A all-atom structure and associated GROMACS itp files were generated using the CHARMM-GUI Glycan Modeler.^53^ d-Glucuronic acid (GLA) subunits were connected to *N*-acetyl-d-galactosamine (GAL) via β 1,4 linkages. Then, GAL was connected to GLA via β 1,3 linkages. The structure was extended in such an alternating fashion until it contained 21 subunits, with 11 GLA and 10 acetyl GAL sugars in total. All *N*-acetyl-d-galactosamines were modified by attaching an O-linked sulfate to the fourth carbon.

### All-Atom MD Simulations of CSA

For comparison, all-atom MD simulations of either a single 21mer CSA chain or four of them were carried out. The CHARMM36m glycan parameters were used for such simulations.^52^ Simulations were carried out in cubic boxes with periodic boundary conditions. For the single-chain simulation, the box size was (9.77 nm)^3^, and for the multichain simulations it was (17.36 nm)^3^. In the latter case, the chains were initially positioned such that at least 1 nm of distance was between them. In both the single-chain and four-chain simulations, the box was solvated with CHARMM-TIP3P water molecules^54,55^ and neutralized with an excess of sodium ions. In addition, NaCl salt up to a final concentration of 0.15 M was added to the systems. The solvated systems consisted of approximately 90,000 atoms (single chain) and 500,000 atoms (four chains). Steric clashes were removed by energy minimization using the steepest descent algorithm. Thermalization followed under NVT conditions for 125 ps of MD at a temperature of 303.15 K. The solvent was subsequently equilibrated around the glycans by performing 250 ps of MD in the NPT ensemble, maintaining the temperature at 303.15 K and the pressure at 1 bar. During minimization, thermalization, and solvent equilibration steps, positional restraints of 400 kJ/mol/nm^2^ on backbone heavy atoms of the glycan rings, 40 kJ/mol/nm^2^ on the side-chain heavy atoms, and 4 kJ/mol/nm^2^ dihedral restraints were imposed. Equilibrium dynamics were then simulated, releasing the positional and dihedral restraints, for five replicates of 1 μs each (5 μs cumulative) for each system.

### Coarse-Grained Simulations of CSA

Coarse-grained simulations of CSA were carried out for a single 21mer, for a system containing four 21mer chains, and for a single 123mer. The coarse-grained models for the single and four 21mers were generated by center-of-geometry mapping of the NPT-equilibrated all-atom structure using the Fast-Forward (ff_map) tool,^56^ retaining the same simulation box dimensions, CSA chain and ion number, as well as their positions. The 123mer chain was built by extending the CG 21mer with PyMOL^57^ and solvated in a (51.72 nm)^3^ box with 0.15 M neutralizing salt, resulting in a total of about 1 million particles. The Martini3 ^48^ coarse-grained force field was used. To avoid CSA chain aggregation, NaCl ions were represented as Q5 beads with ±0.75 charge, unless otherwise noted. The bead type Q5 accounts for the hydration shell around the ions. Furthermore, charge rescaling of ± 0.75 corresponds to the proposed correction to take into account the electronic polarization of water^58^ (see the section CSA Interchain Interactions for a detailed explanation). Martini 3 water (representing four atomistic water molecules in one bead) was used to solvate the system. Files containing the initial interaction parameters (itp) suitable for GROMACS were generated with Polyply (gen_params).^59^ For simulations with noninteger salt ion charges, additional ions were added until the system was fully neutralized. Energy minimization was carried out to remove steric clashes for 2000 steps using the steepest descent algorithm, followed by equilibration in the NPT ensemble, for 75 ns. Finally, five production runs of 1 μs were performed for simulations with 21mers and 10 runs of 3 μs for the 123mer.

### Simulations of VAR2CSA in Complex with CSA

Equilibrium coarse-grained MD simulations of the core domain of VAR2CSA (NF54 strain) in complex with a fully sulfated CSA 21mer were carried out. The conformations of the complex were retrieved from previous all-atom equilibrium MD simulations^40^ (at a time point 150 ns of each of the 10 replicas). The CSA chain was coarse-grained following the same process outlined above. The Martini 3 model of VAR2CSA was built using Martinize2 choosing default parameters,^60^ including secondary structures predicted by DSSP^61^ and native contacts.^62^ In addition, to preserve the tertiary structure of the protein, a Go̅ model, establishing a network of pairwise Lennard-Jones interactions of strength ϵ_LJ_ = 8.414 kJ/mol, was applied.^63,64^ Go̅ interactions were truncated at a distance lower than 0.3 nm or greater than 1.1 nm and were excluded from the internally disordered region spanning residues from 376 to 551. To remove steric clashes, the complex was energy-minimized using the steepest descent for 100 steps. The energy-minimized complex was placed in a dodecahedral box, with periodic boundary walls at a distance of at least 1 nm from the complex, and solvated with Martini 3 water. The system was neutralized at an ionic strength of 0.15 M NaCl, with both Na and Cl represented as Q5 beads of ±0.75 e charge. The noninteger excess charge was controlled by adding a single TP1 bead with a charge of −0.25 e, which is not expected to introduce any significant interactions in the system. Energy minimization and three 750 ps rounds of NPT equilibration preceded the production runs. During the first round, position restraints (with elastic constant of 1000 kJ/mol/nm^2^) were applied to all protein and glycan beads. During the second round, only protein beads were restrained. Finally, the third round was performed without any restraints. Production runs of 300 ns were subsequently performed independently for 10 replicates.

The final conformation after 300 ns of equilibration was used as the starting configuration for force-probe MD simulations. Virtual harmonic springs (1000 kJ/mol/nm^2^ force constant) were attached to the C-terminal methionine 1953 of VAR2CSA and the first monomer of the CSA 21mer chain and moved with a constant velocity of 0.1 m/s in opposite directions. *N* = 10 force-probe MD simulations were carried out, each lasting 120 ns.

### MD Simulation Parameters and Algorithms

All-atom MD simulations were performed using the GROMACS MD package (version 2025.2).^65^ Newton’s equations of motion were numerically integrated at time steps of 2 fs (1 fs during thermalization) using the leapfrog algorithm. The temperature (pressure) was kept constant by coupling the simulated system to the Nose–Hoover thermostat (Parrinello–Rahman barostat), with coupling constants of 1 ps (5 ps) every 500 (2500) integration steps. Short-range nonbonded van der Waals interactions were modeled by a Lennard-Jones potential truncated at a distance of 1.2 nm. Electrostatic interactions were treated using the particle mesh Ewald (PME) algorithm.^66,67^ Neighbors were considered via the Verlet buffer scheme^68^ with a tolerance of 0.005 kJ mol^–1^ps^–1^. LINCS^69^ was used to constraint bonds involving heavy atoms of the sugars, while SETTLE^70^ was employed to constraint both bonds and angular vibrations of water molecules.

Coarse-grained Martini 3 MD simulations were carried out using the same parameters and algorithms as for the all-atom simulations, except for the following differences. The integration time step was 15 fs. Temperature–pressure coupling was achieved with Berendsen thermostat and barostat^71^ during minimization or equilibration and with the v-rescale^72^ (Parrinello–Rahman^73^) during production. The temperature coupling constants were 1, 2, and 1 ps during minimization, equilibration, and production, respectively. Meanwhile, the pressure coupling constant was 12 ps during all steps. Instead of a tolerance in the interactions with the Verlet buffer neighbor scheme, a fixed neighbor list cutoff of 1.35 nm was used.^74^ Electrostatic interactions were treated either with the reaction-field scheme^75^ (dielectric constant: 15; cutoff radius: 1.1 nm) or with PME for the simulations of 21mers, and exclusively with PME for the simulations of the 123mer or a CSA 21mer in complex with VAR2CSA.

### Analysis

The solvent-accessible surface area (SASA) was determined using the double cubic lattice method as implemented in the GROMACS *gmx sasa* tool,^76^ using a probe sphere of size 0.191 nm. Atomic van der Walls radii were retrieved from,^77^ and for the Martini 3 beads the following sizes were used: 0.264 nm for regular, 0.23 nm for small, and 0.191 nm for tiny beads.^48^

Center-of-geometry mapping of the all-atom trajectory to Martini 3 beads and quantification of bonded term distributions were carried out by using Fast-Forward.^56^ The bonded term distributions were compared by means of the Jensen–Shannon divergence score, available in the SciPy library.^78^ The optimal bin width for each bonded term comparison was selected using the Freedman–Diaconis rule.^79^

The GROMACS *gmx polystat* function was used to compute both the end-to-end distance and the radius of gyration of the CSA chains. *gmx distance* was employed for computing pairwise residue distances, defined as the distance between virtual sites located at the center of each sugar ring. It should be noted that such a criterion is the most appropriate as it depends only on the positions of the sugar rings and is not influenced by the orientation of functional groups. *gmx distance* was also used to quantify protein–CSA contacts by considering two beads “in contact” if their minimum distance was less than 0.45 nm. The radial distribution function of sodium was calculated using *gmx rdf*, with either the carboxylic acid or sulfate group bead acting as the reference particle and the sodium bead acting as the selection. Root-mean-square fluctuations (RMSFs) of the protein residues or CSA monomers were determined with *gmx rmsf*.^65^ Unless otherwise stated, persistence length was defined as the exponential decay half-life of the GLA virtual site autocorrelation function *C*(*x*), quantified with *MDAnalysis.analysis.polymer.PersistenceLength*.^80^ *MDAnalysis.analysis.rms.rmsd* ^81,82^ was used to calculate the VAR2CSA root-mean-square deviation.

The Flory scaling exponent ν was determined by fitting the function ⟨*d*_*ij*_⟩ = *b*|*i* – *j*|^ν^ to the curve ⟨*d*_*ij*_⟩ versus |*i* – *j*|, where ⟨*d*_*ij*_⟩ is the average distance between residues *i* and *j*, and *b* is the proportionality constant.^83^

For VAR2CSA simulations, analysis was computed for each replicate from 150 to 200 ns time points in all-atom trajectory and from 100 to 300 ns for coarse-grained to account for equilibration. Analysis of the CSA 123mer trajectory was done from 500 to 3000 ns for the same reason.

Molecular structures were visualized and rendered with VMD.^84^

The CG force-field parameters, MD simulation parameters, and initial configurations generated in this study are deposited at https://github.com/PaulGreic/Csa_Martini3 and submitted to the Polyply library.

## Results

### Parametrization of the Repeating Unit

CSA is a linear polysaccharide composed of repeating d-glucuronic acid (GLA) and *N*-acetyl-d-galactosamine 4-sulfate (GAL) units (Figure 1A,B). We built a Martini 3 coarse-grained model of this repeating unit, validating it against all-atom simulations using the CHARMM36m force field. Five × 1 μs replicas of CSA 21mer all-atom simulations were sufficient to ensure a converged reference data set (Figure S14). Most of the model was based on existing atom-to-bead definitions from other carbohydrate and small-molecule models (Figure 1C).^49,85^ The sulfate group, which is of key importance for CSA, has not yet been parametrized in Martini 3. We mapped it following general force-field guidelines: four heavy atoms, including a period three-element sulfur, were represented as a regular-size bead of diameter 0.264 nm with a Q4n label to account for the −1 charge delocalized among the oxygens.^48^ In addition, in order to capture the ring-stacking interactions, as recommended for Martini3 carbohydrates,^49^ a virtual interaction site was added to the center of each six-membered glycan ring. The resulting model mapped 53 atoms into 11 coarse-grained particles.

As in other Martini 3 carbohydrate models, to capture the rigid nature of the glycan rings, beads representing them were connected via distance constraints rather than harmonic bonds.^49^ Furthermore, the distance between such beads was increased by 10% compared to the atomistic reference to more accurately represent the molecular volume (Figure S1). This resulted in a distribution of solvent-accessible surface area (SASA) for our Martini 3 model that compared extremely well to that distribution for the atomistic reference (Figure 2A), a result that was visually corroborated by the Connolly surfaces (Figure 2B).Thus, the coarse-grained model adopted here accurately captures the shape of the CSA repeating unit.

**Figure 2:**
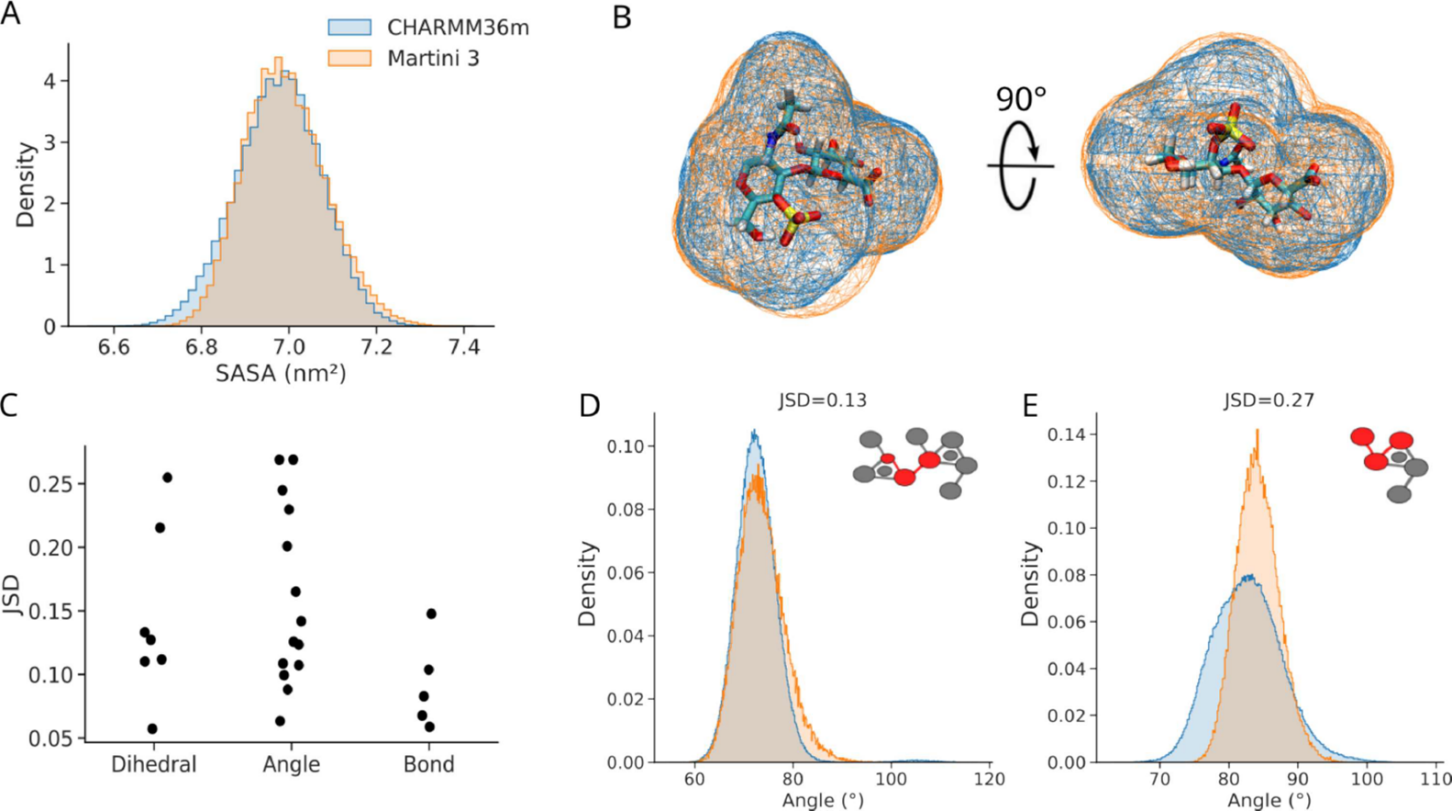
CG Martini 3 model of CSA accurately reproduces its exposure to the solvent and bonded term distributions. (A, B) Solvent-accessible surface area (SASA) distribution (A) and Connolly surface (B) comparison for the GLA–GAL repeating unit of CSA, recovered from MD simulations at all-atom (CHARMM36m) and coarse-grained (Martini 3) resolution. (C) Jensen–Shannon divergence statistic for the three types of bonded terms used, comparing the Martini 3 model with the all-atom CHARMM36m reference data (JSD = 0: identical distributions, and JSD large: dissimilar distributions). (D, E) Examples of bonded interaction distributions from coarse-grained and forward-mapped all-atom simulations for the bonded term marked in red in the inset: one close to the median, representative of a low value of the JSD (D), and one with the largest JSD, representing the worst agreement (E). B, D, and E follow the same color scheme as A. See the comparison of distributions for all bonded terms in Figures S2–S4.

All other bonded interactions within the disaccharide repeating unit were adjusted to match their corresponding distributions in all-atom simulations. It should be noted that in order to make the comparison possible, all-atom trajectories were forward-mapped based on the center-of-geometry of atom groups corresponding to each Martini bead. The resulting distributions are shown in Figures S2–S4. The agreement between Martini and reference (all-atom) distributions of each bonded term was quantified using the Jensen–Shannon divergence (JSD), which gives a score of 0 for identical distributions and a score of 1 for nonoverlapping distributions (Figure 2C). The median JSD value was 0.13, indicating that most of the terms were well reproduced by the coarse-grained model (Figure 2C,D). The worst JSD value (0.27) was observed for an angle with an asymmetrical reference distribution, which is a difficult case to reproduce at this level of resolution (Figure 2E). Similarly, dihedral angles that span across a single glycosidic bond contained minor peaks in the reference distributions that were not captured during Martini 3 simulations (Figure S4). Analysis of the all-atom trajectories revealed that these peaks resulted from nonexosyn conformations of the glycosidic bond (Figure S11), indicating that the coarse-grained CSA model is unable to capture anti-ϕ or anti-ψ conformers. It should be noted that the coarse-grained simulations were run using modified parameters to avoid CSA aggregation (see below); nevertheless, similar results were also achieved with default Martini 3 parameters (Figure S5A).

### Evaluation of Polymer Properties

After establishing a Martini 3 model for the CSA repeating unit, we evaluated whether it is capable of reproducing the atomistic polymer properties of a CSA 21mer under equilibrium conditions (Figure 3A). The size of polymers is commonly quantified with the end-to-end distance (ETE) or the radius of gyration (*R*_g_). For a Gaussian chain, the ratio ⟨ETE^2^⟩/⟨*R*_g_^2^⟩ ≈ 6.^86^ In the reference all-atom simulations, the ratio was 10.4, indicating that this glycan chain adopts a highly extended conformation. Reassuringly, a similar level of elongation was observed in our coarse-grained Martini 3 model, where the ratio was estimated to be around 10.0 (Figure 3B). Furthermore, the characteristic ratio, i.e. ⟨ETE^2^⟩ /(*Nb*_v_^2^) (with *N* = 21, the polymerization degree, and *b*_v_ = 0.52 nm, the bond length), was found to be 10.8, which is consistent with the estimate from a previous implicit-solvent CG model of CSA (see the value of ∼10 for comparable *N* in Figure 6 in Bathe et al.^43^).

**Figure 3:**
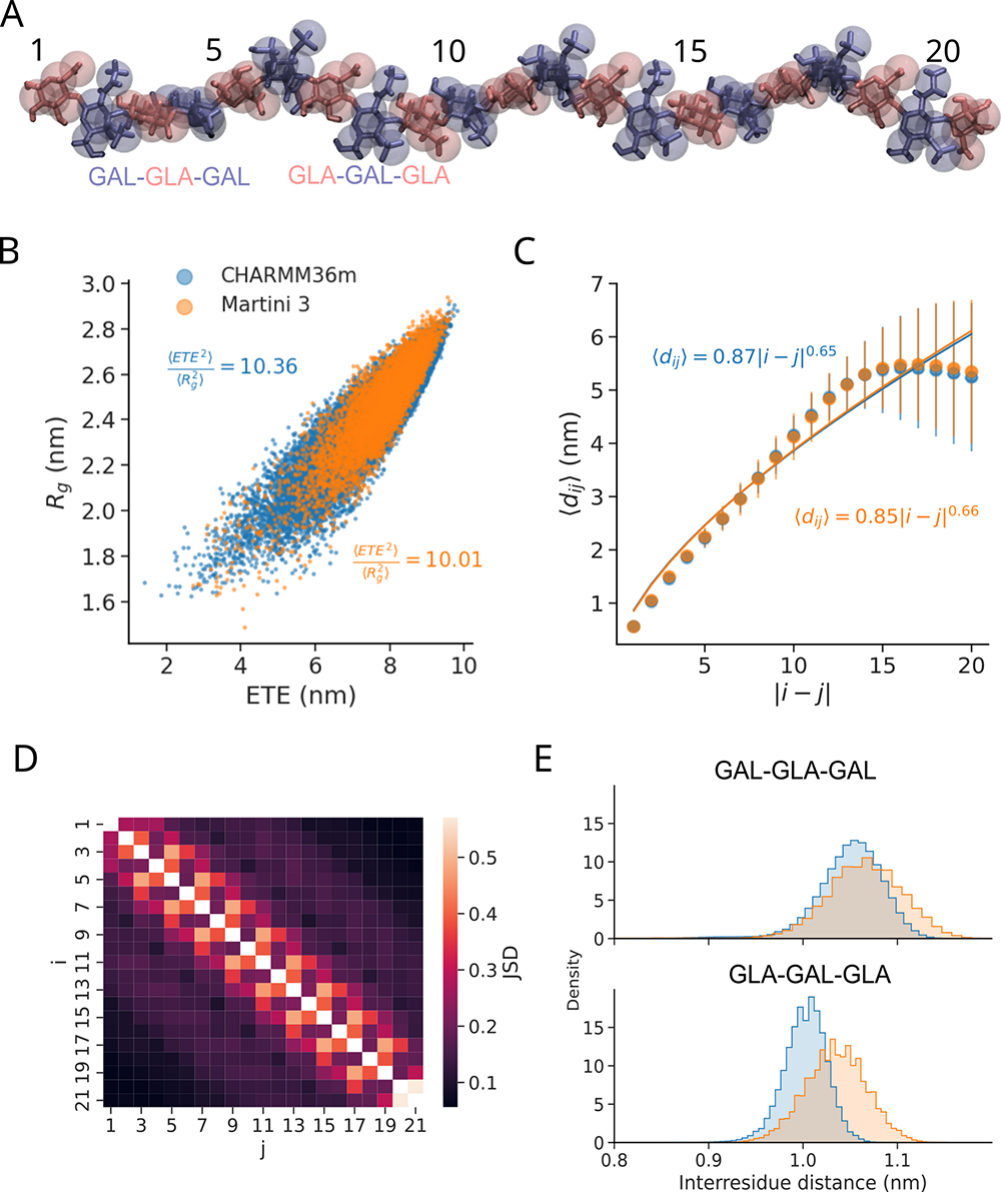
Coarse-grained Martini 3 model captures the polymer behavior of a single CSA chain. (A) A 21-saccharide CSA chain, superposing the all-atom structure (sticks) with the CG Martini 3 model (spheres) highlights the alternating nature of the GAL and GLA units. (B) Ratio between the radius of gyration *R*_g_ and the end-to-end distance ETE, recovered from MD simulations using all-atom (CHARMM36m) and Martini 3 models (color). The average squared ratio ⟨ETE^2^⟩/⟨*R*_g_^2^⟩ is indicated in both cases. (C) Average inter-residue distance ⟨*d*_*ij*_⟩ between the *i*-th and the *j*-th saccharide of the chain (scatterplot: av. ± s.e.). The line shows the fit ⟨*d*_*ij*_⟩ = *b*|*i* – *j*|^ν^, where *b* is the proportionality constant (in nm) and ν is the scaling exponent. The resulting fitting parameters are indicated for both studied cases. (D) Jensen–Shannon divergence (JSD) of intersaccharide distance distributions between all-atom and coarse-grained models (JSD = 0: identical distributions, and JSD large: dissimilar distributions). (E) Intersaccharide distance when |*i* – *j*| = 3 for two possible fragments, GAL–GLA–GAL and GLA–GAL–GLA (see A). C and E follow the same color scheme as B.

Polymers are also highly influenced by the surrounding solvent. To confirm that the balance between polymer–polymer and polymer–solvent interactions was maintained in our Martini 3 CSA system, we quantified the Flory scaling exponent, ν, from the distance *d*_*ij*_ between saccharides *i* and *j* along the CSA chain (Figure 3C). Our coarse-grained model reproduced almost perfectly the distances observed in the all-atom case. More specifically, the CSA scaling exponent obtained from distances was very close in the two descriptions, i.e., 0.65 for the all-atom and 0.66 for the coarse-grained model. From polymer theory, under equal contributions of polymer self-interactions and polymer–solvent interactions, ν = 0.5, while ν > 0.5 indicates dominance of protein–solvent interactions, rendering a highly solvated and ‘’swollen” chain.^83^ Our results indicate the latter to be the case for the studied (21 monomer long) CSA chain.

Lastly, we inspected the pairwise distances between CSA chain units by also comparing the distributions using Jensen–Shannon divergence (Figure 3D). We found that when |*i* – *j*| >3, the JSD score did not exceed 0.25, indicating that at larger length scales there is high concordance between CG and AA models. However, comparison of closer saccharide pairs revealed that the distributions between GLA and its neighbors had a larger divergence. In particular, comparison of two neighboring GLA residues (GLA–GAL–GLA) resulted in the worst JSD score of 0.48, caused by the CG distance being overestimated by 0.03 nm on average (Figure 3E, bottom panel). In contrast, the JSD score for neighboring GAL residues was 0.22, indicating good overlap between coarse-grained and all-atom distributions (Figure 3D,E, top panel). Because the error in pairwise distances did not propagate along the chain (Figure S6) and did not affect chain stiffness (Figure S10), we deemed it acceptable given the resolution of Martini 3 models.

In summary, our CG Martini model reproduces well the conformational properties and the balance between glycan–glycan and glycan–solvent interactions of a short CSA chain. It should be noted that comparable behavior was also observed when CG simulations were performed with the original Martini 3 parameters (Figure S5B–D), but as in the case of the disaccharide, we showed here the results for the optimized parameters that did not lead to chain aggregation, as explained in the following section.

### CSA Interchain Interactions

It is not only single chains in isolation but also multiple coexisting chains that are the topic of interest in CSA research. Accordingly, a key requirement for our coarse-grained model was to ensure that chains remained well solvated and dispersed. In order to check this, we carried out simulations of a mixture containing four 21mer chains (corresponding to a molar concentration of 1.27 mM) and monitored their aggregation tendency. The level of aggregation was quantified as the ratio between the SASA of all chains together, *S*_tot_, and the sum of the SASA of each chain, ∑_*a* = 1_^4^ *S*_*a*_. This ratio equals one if the chains are not in contact (no aggregation) and tends to zero if the chains get in contact and thus aggregate. Due to the strong electrostatic repulsion between the negatively charged sulfate and carboxylic acid groups, at an all-atom resolution, on the microsecond time scale, the chains repelled each other not showing any sign of aggregation (see data for CHARMM36m in Figure 4A,B). On the contrary, coarse-grained simulations using the default Martini 3 parameters revealed a strong aggregation tendency (see Martini 3, Reaction-field, *q*(*ions*) = ±1.0, tiny (TQ5) case in Figure 4B). Calculation of radial distribution functions demonstrated that, in this case, the sodium density around negatively charged CSA groups was found to be much higher than in the all-atom system (Figure S6A). We also noted that these cations can get sandwiched among negatively charged CSA beads, thereby bridging the distance between CSA chains (Figure S6B). Thus, we explored whether such observed interchain association could be prevented by reducing the strength of the interaction between sodium and the anionic CSA moieties.

**Figure 4:**
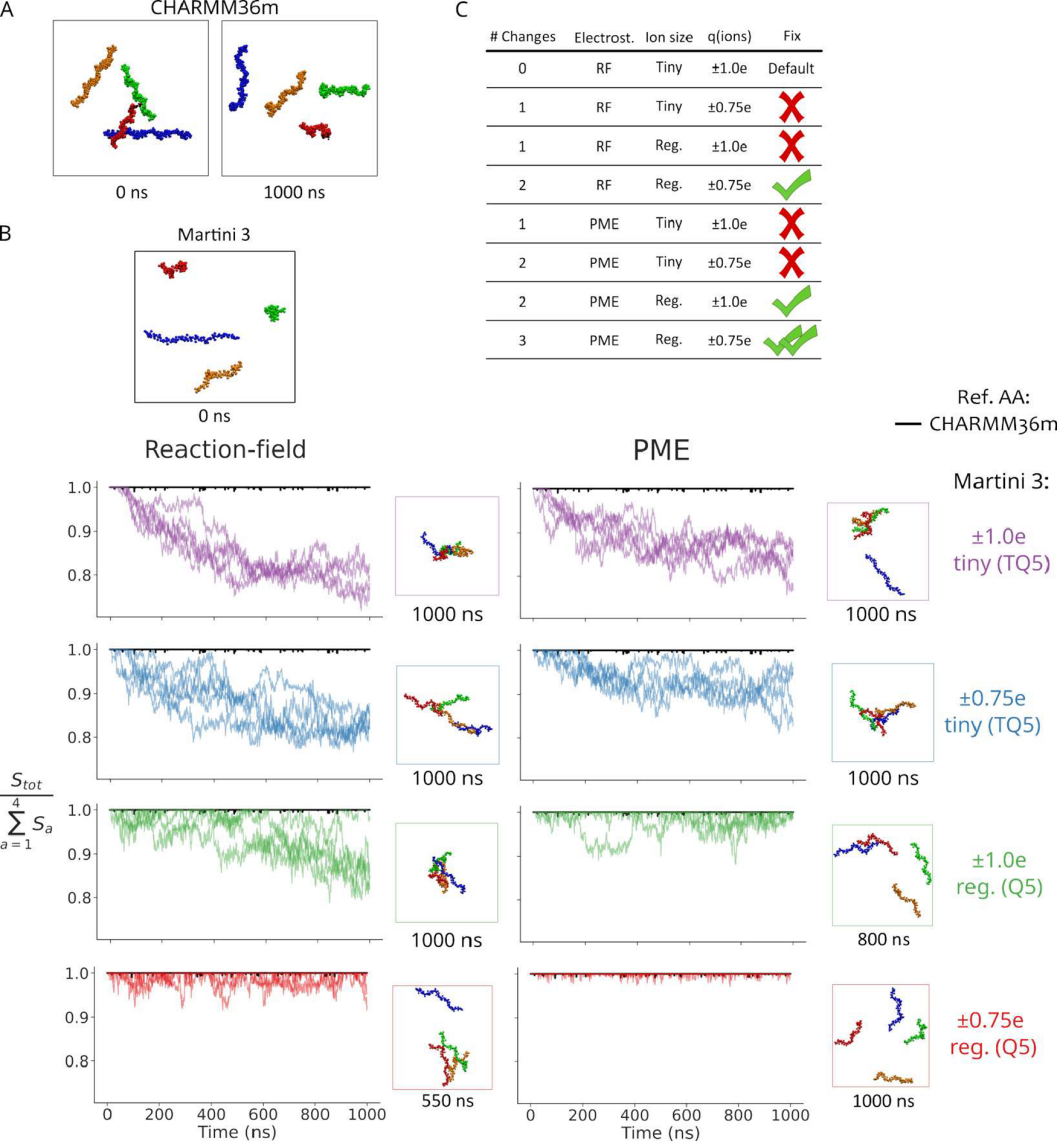
Electrostatics and ionic strength control CSA aggregation in Martini 3. (A) Snapshots extracted from all-atom (CHARMM36m) simulations of a 4xCSA 21mer system. (B) At the top, a snapshot of said system, simulated under different CG setups, is shown. At the bottom, time traces of the chain exposure ratio are presented (*n* = 5). ∑_*a* = 1_^4^ *S*_*a*_ = 1 corresponds to no chain aggregation. The lower this ratio, the higher the aggregation. The electrostatic treatment (reaction-field or PME), sodium and chloride ion bead size (tiny TQ5 or regular Q5), and the ionic charge (*q* = ±1.0 e, or *q* = ±0.75 e) were systematically varied for Martini 3 simulations. The default condition used in Martini 3 corresponds to the reaction-field, with tiny (TQ5) sodium and chloride beads and ionic charges of *q* = ±1.0 e (top-left panel, purple). Next to the time traces, a snapshot from each simulation at the indicated time is shown. Time traces for atomistic simulations are shown in black as a reference. In this case, the exposure ratio was almost 1, in the studied time scale, indicative of no aggregation. (C) Summary of studied conditions, indicating to which extent they fixed aggregation. Individual CSA chains in simulations snapshots are shown in different colors (red, blue, green, and yellow).

Three aspects of relevance to the interaction of glycans with ions were considered. First, overestimation of Coulomb interactions is a common issue among nonpolarizable force fields.^58^ One strategy to address this problem is by rescaling charges.^87^ The proposed rescaling factor is proportional to the electronic polarization contribution of water screening,^58^ which would result in a reduction of monovalent salt charges from ±1.0 to ±0.75. We tested such a correction in our system by applying it to sodium and chloride ions. To only minimally disturb the Martini 3 force field, other charges in the system were not rescaled. In particular, because a charge of −1.0 e and SQ5n beads have been used to represent carboxylic acid moieties in various other Martini models, we opted not to rescale this charge either. Second, the Coulomb potential in Martini 3 is usually handled using the reaction-field scheme.^48^ Although less computationally intensive, this method can lead to artifacts, including truncation and discontinuities of the potential function and a mean-field approximation of long-range electrostatics.^88^ Thus, we tested particle mesh Ewald (PME), which is an alternative. PME alleviates these issues at the expense of a higher computational cost. PME is the standard in all-atom simulations, although it has also been used in Martini coarse-grained simulations, e.g., of an ionic liquid.^89^ Lastly, a hydration shell forms around ions suspended in water. In older versions of Martini, this was implicitly accounted for by representing ions with bead sizes larger than their ionic radius.^90^ However, this practice was abandoned in Martini 3, resorting to the use of the smallest bead, TQ5. The choice of bead radius would not only influence the size of the ion particle but also affect its Lenard-Jones potential parameters. For this reason, we explored the effect of using a larger bead for monovalent ions (i.e., Q5). Consequently, we conducted equilibrium simulations of the 21mer CSA chain mixture with all possible combinations of these three conditions to test how they influenced interchain association.

As mentioned, the default Martini 3 simulation setup (i.e., RF treatment, tiny TQ5 beads of *q* ± 1.0 e for sodium and chloride) showed the largest decrease in the exposed area ratio, implying the largest level of aggregation (Figure 4B, top left). Changing each of the three above-mentioned aspects individually reduced aggregation, however, not fully (Figure 4B). For RF versus PME, compare the left-column and right-column purple curves; for bead size, compare the tiny bead (purple) and the regular bead (green) curves; and for charge scaling, compare *q* = ±1.0 e (purple) with *q* = ±0.75 e (blue). The situation improved when two conditions were adjusted at the same time. In two simulation setups (either RF with regular bead size (Q5) and rescaled charge or PME with regular bead size (Q5) and full *q* = ±1.0 e charge), the exposure ratio fluctuated around one, indicating the formation of brief and unstable small aggregates (Figure 4B). Interestingly, using PME and rescaling the ionic charges, while still describing sodium with a tiny bead, did not show significant improvement. Finally, aggregation was fully avoided, as observed in all-atom simulations, when all three conditions, i.e., PME, (Q5) regular-size beads, and rescaled ionic charge of *q* = ±0.75 e, were in place (Figure 4B).

To test how these changes might alter single-chain polymer properties, we also conducted equilibrium simulations of a single CSA 21mer chain and evaluated the end-to-end distance and radius of gyration for each case (Figure S12). In the case corresponding to PME treatment with tiny TQ5 beads of *q* ± 1.0 e for sodium and chloride, the polymer displayed collapsed conformations that were not seen in other CG simulations nor in the all-atom reference ones. Apart from this case, the polymer conformation was similar in all other considered schemes, most importantly in the three nonaggregating ones. We also evaluated the RDFs in these simulations, demonstrating that the proposed changes indeed reduced the cation distribution around anionic groups, although still not to the level of the all-atom reference (Figure S13).

In summary, we identify at least three distinct possible treatments of glycan-ion electrostatic interactions that give satisfactory aggregation levels of CSA at coarse-grained resolution (Figure 4C). To illustrate the applicability of our coarse-grained model in two relevant contexts, in the following we consider the conditions that led to full abolishment of aggregation, i.e., after using PME and regular-size beads for sodium or chloride ions with rescaled charges.

### CSA in Complex with VAR2CSA

After establishing a coarse-grained model for CSA under nonaggregating simulation conditions, we sought to confirm that such a setup can accurately capture the dynamics of a protein in complex with the glycan. To test this, we chose the malaria adhesin VAR2CSA, which is known to bind CSA chains in their fully sulfated form.^23,24,91^ The interaction of VAR2CSA with CSA is essential for the attachment of malaria-infected erythrocytes to the placenta proteoglycan matrix, leading to pregnancy-associated malaria.^92^ Hence, disruption of this interaction is a promising therapeutic approach for the design of antimalarial vaccines.^93^ This complex has previously been investigated with all-atom simulations.^38,40^ Thus, we here attempted to reproduce the (all-atom) equilibrium dynamics of this complex using our Martini 3 model for CSA. The coarse-grained model of VAR2CSA was generated following standard Martini 3 procedures by applying Go̅-type additional interactions (see Methods).^64^ The initial conformation of the complex at both levels of resolution is illustrated in Figure 5A. The dynamics of the complex were then simulated in *n* = 10 independent replicas of 300 ns each.

**Figure 5:**
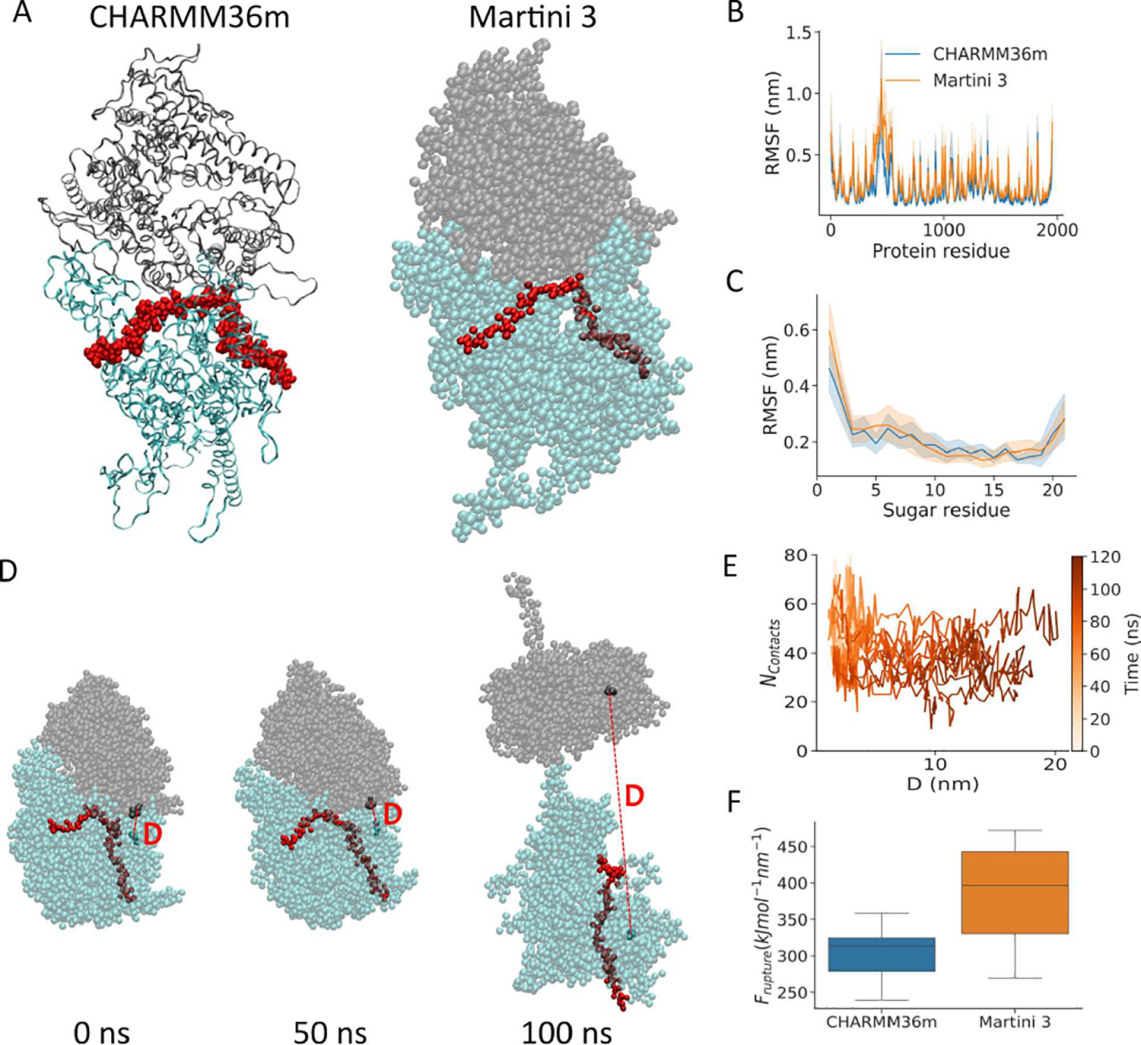
Coarse-grained simulations of CSA bound to malaria adhesin VAR2CSA. (A) Snapshot of VAR2CSA bound to a CSA 21mer (red) in all-atom and coarse-grained Martini 3 resolution. VAR2CSA N-terminal subunit (residues 1–964) is marked in cyan and the C-terminal subunit (residues 965–1954) in gray. (B,C) Root-mean-square fluctuation (RMSF) of the protein backbone (B) and the CSA sugar chain (C) obtained from equilibrium MD simulations. All-atom simulations taken from.^40^ (D–F) Coarse-grained pulling simulations of VAR2CSA. Representative snapshots during the opening process are depicted in part D (the same color code as in part A). Residues used to measure the distance between the N- and C-terminal subunits (red dashed line) were 120 and 1663, respectively (opaque). (E) Number of contacts between VAR2CSA and CSA as the distance between the N- and C-terminal subunits changes. (F) Force needed for unfolding. B, C, E, and F show data from *n* = 10 independent replicates.

As expected, by imposing Go̅ distance restraints, the protein largely retained its structural fold (Figure S7). Local dynamics were subsequently compared using per-residue backbone root-mean-square fluctuations (RMSFs) (Figure 5B). There was good agreement in residue flexibility, with only deviations arising from highly mobile regions. When monitoring the local flexibility of the (bound) CSA chain via RMSF, we also observed good agreement between all-atom and coarse-grained simulations (Figure 5C). Saccharides 3 to 19 were directly in contact with VAR2CSA and thus displayed reduced flexibility, compared to chain ends, which were not in contact and were hence more mobile. The high agreement of RMSF values demonstrates that the equilibrium dynamics of CSA in complex with a protein can be accurately reproduced by our glycan model in conjunction with a conventional protein model used for Martini.

Previous atomistic simulations have also demonstrated that applying elongational tension to VAR2CSA causes an opening of the core region,^40^ a process of relevance for the shear-enhanced adherence mechanism of malaria-infected erythrocytes mediated by this protein.^94^ We thus checked whether our CG model was also capable to capture the force response of this complex. By performing constant-velocity force-probe MD simulations, applying the same elongational tension on the C-terminal methionine and the 21st sugar residue of the CSA chain, we were able to achieve the same type of opening behavior (Figure 5D). The distance between the two subdomain regions suddenly increased upon surpassing a certain force threshold (*F*_rupture_) value, and the opening always preceded glycan dissociation (Figure 5E). We quantified the rupture force by identifying the time point when the VAR2CSA interdomain distance was ≥5 nm and then identifying the maximal pulling force within a 10 ns window around this time point. *F*_rupture_ in CG simulations was greater than in all-atom simulations, likely because the default Go̅ restraints were acting on the opened interface, creating additional resistance not present in the atomistic protein model (Figure 5F). However, the difference was not substantial, and in agreement with all-atom simulations, the force required to open the VAR2CSA core region was lower than the force needed for CSA dissociation; thus, opening always preceded dissociation (Figure 5E,F). Thus, not only under equilibrium but also under nonequilibrium tension conditions, our coarse-grained model of CSA (together with a Go̅-type protein description) properly captures the specific properties of the VAR2CSA–CSA complex.

### Simulations of a CSA 123mer

So far, we have focused our analysis on 21mer CSA chains to allow comparison with all-atom simulations and demonstrated that our coarse-grained model accurately reproduces the dynamics of atomistic CHARMM36m simulations. However, in vivo CSA chains are often longer than 100 saccharides—a scale which is no longer feasible to simulate at an all-atom resolution. Therefore, we proceeded to simulate a CSA 123mer by using our CG Martini 3 model for 10 independent replicates of 3 μs each (Figure 6A and Movie S1). Surprisingly, although the energy surface of the coarse-grained resolution was smoother, these simulations revealed slow equilibrium dynamics, as reflected by slow, gradual fluctuations of the end-to-end distance (Figure S9). Thus, not only does the Martini 3 model of CSA enable simulations of more biologically relevant size scales but it also allowed us to capture conformational states that would take much longer to sample atomistically.

**Figure 6:**
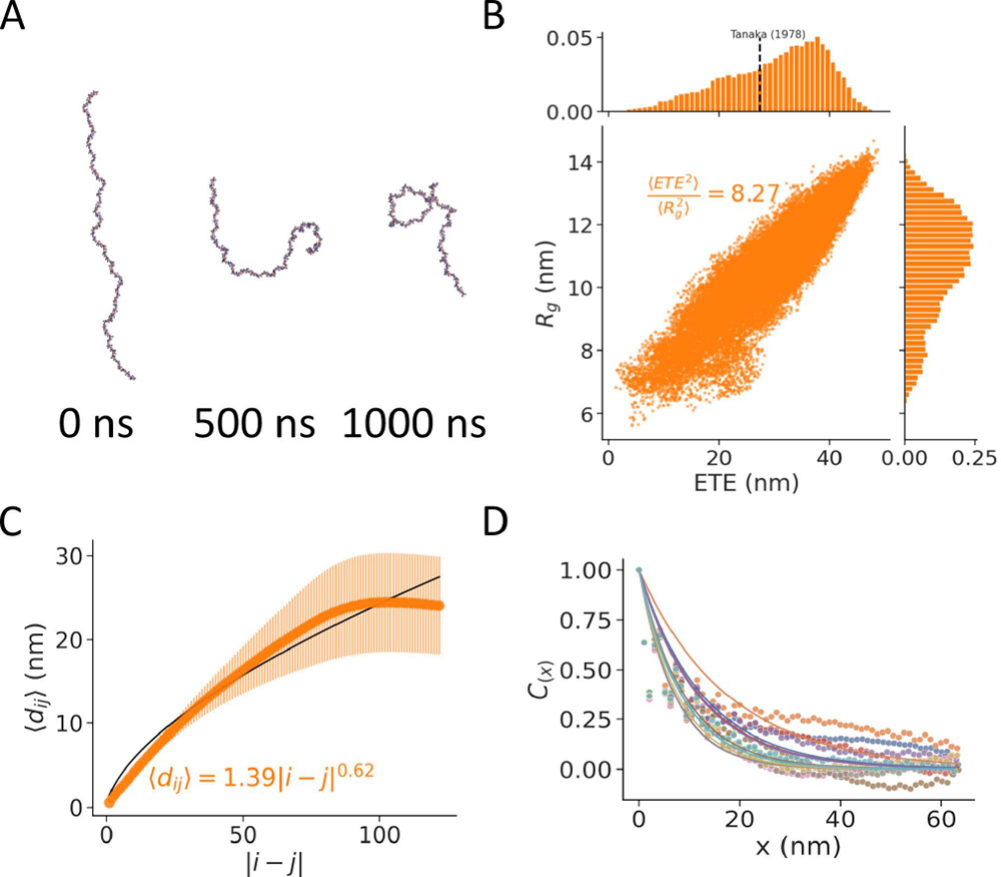
Coarse-grained CSA 123mer simulations. (A) Snapshots of a 123-sugar CSA chain in CG resolution at different time points of the equilibrium simulation (GLA: red and GAL: blue). (B) Ratio between the radius of gyration *R*_g_ and the end-to-end distance ETE and their distributions, recovered from MD simulations. The text indicates the average squared ratio, ⟨ETE^2^⟩/⟨*R*_g_^2^⟩. The dashed line indicates the experimental mean root-squared end-to-end distance.^13^ (C) Average inter-residue distance ⟨*d*_*ij*_⟩ between the i-th and the j-th monomer of the chain (scatterplot: av. ± s.e.). The black line shows the fit ⟨*d*_*ij*_⟩ = *b*|*i* – *j*|^ν^, where *b* is the proportionality constant, 1.39 nm, and ν is the scaling exponent, 0.61. (D) Decay of the autocorrelation *C*(*x*) of GLA virtual sites with distance *x* (simulation data: points; exponential decay fit: lines). The distance at which *C*(*x*) = 0.5 was considered the persistence length. Different colors represent *n* = 10 independent replicates.

The simulations of the 123mer allowed us to quantify the polymer properties of a fully sulfated CSA chain in explicit solvent. Consistent with the CSA 21mer, we find that the ratio ⟨ETE^2^⟩/⟨*R*_g_^2^⟩ > 6 pointed to an extended conformation (Figure 6B). In addition, the mean end-to-end distance of this chain was 30 nm, which is close to the experimentally measured value for a chain of a similar molecular weight of 27.3 nm,^13^ while previous estimates with an implicit-solvent CG model^43^ predicted values of the order of 32.4 nm. The scaling exponent is similar to our estimate for a CSA 21mer, confirming the dominance of the polymer–solvent interactions (Figure 6C). In addition, the characteristic ratio, i.e., ⟨ETE^2^⟩ /(*Nb*_v_^2^) (with *N* = 123, the polymerization degree, and *b*_v_ = 0.52 nm, the bond length), was found to be 27, which falls in the same range of a previous estimate from an implicit-solvent CG model (between ∼30–35 for a comparable *N* in Figure 6 in Bathe et al.^43^). Previous studies using implicit-solvent CG simulations and dynamic light scattering experiments reported hydrodynamic radii, *R*_h_, values of around 5.5 nm, for CS chains of comparable polymerization degree,^45^ while explicit-solvent CG simulations (using Martini 2.2) reported values of ∼4.8 ± 0.3 nm for a 100-monomer fully solvated chain.^47^ Assuming a linear monodisperse chain, *R*_g_ = 1.5 *R*_h_,^45,95^ our estimate of the radius of gyration, *R*_g_ = 10.8 ± 1.6 nm (mean ± s.d., Figure 6B), turns into an *R*_h_ = 7.2 ± 1.1 nm. Although larger, our estimate is not too far from such previous estimates, especially taking into account the differences in the samples (i.e., size and sulfation distribution), environmental conditions, and the approximate nature of the *R*_g_ to *R*_h_ conversion.

Lastly, we estimated the persistence length (*l*_p_) of 10.3 ± 3.2 nm (average ± s.d.) according to the exponential decay half-life of the autocorrelation function *C*(*x*) (Figure 6D). The large variation among replicates is attributed to the slow dynamics of the chain. Therefore, we also quantified *l*_*p*_ using an alternative method, assuming the CSA chain to be an idealized worm-like chain (WLC). For a WLC with contour length (*L*) greater than *l*_p_, the following relationship holds: ⟨ETE^2^⟩ ≈ 2*l*_p_ *L*.^95^ Using ETE at *t* = 0 as the contour length of the 123mer chain (50.1–51.9 nm), *l*_p_ was 9.6 ± 2.1 nm. Encouragingly, both estimates are not far from previous computational persistence length estimates, which saturated at 11.2–12.0 nm for several hundreds, i.e., *N* = 256, chains^43^ and from a small angle neutron scattering estimate of 12.7 nm (for a 0.01 w/w concentration).^96^

## Discussion

Chondroitin sulfate A (CSA) is a linear glycosaminoglycan that is attached to cell surface or secreted proteins.^1^ It regulates humoral signaling pathways,^17^ ensures hydration and toughness of various tissues,^10–12^ acts as an antioxidant,^15^ and is implicated in many other vital roles. Research into CSA has been difficult due to its large size, heterogeneous chemical composition, and multitude of binding partners. Here, we developed a model of CSA for coarse-grained simulations based on the Martini 3 force field to address these challenges.

Previous high-resolution simulations of CSA have been limited to short fragments.^31–33,35^ In our CG model, the 53 atoms constituting the disaccharide repeating unit are represented by 11 Martini particles. Despite this reduction in the level of detail, our model still retained an accurate representation of the repeating unit’s molecular volume and its internal bonded interactions (Figure 2). Subsequently, the parameters of the repeating unit could be used to build longer CSA chains. For medium-sized chains, of a couple tens of monomers, our results compare extremely well with atomistic simulations (performed using the CHARMM36m force field^52^) in terms of description of polymeric properties, such as the chain extension and intermonomer interactions (Figure 3). The minimal CSA segment that binds to the malarial adhesin VAR2CSA has such a degree of polymerization,^24^ and we thus show that our CG model is capable of capturing the key structural details of CSA fragments of that length.

Beyond short fragments, the modularity of the model allowed investigation of the behavior of much longer CSA chains. The degree of polymerization of this glycan in nature is of the order of several tens or even hundreds of monomers.^97^ For a chain in that range (exactly 123 monomers), we predict structural properties that are encouragingly close to experimental estimates^13,45,96^ and to previous estimates from implicit-solvent^43,45^ and explicit-solvent^47^ CG models (Figure 6). It should be noted that differences in the exact size and sulfation distribution, as well as in buffer conditions, make this comparison not straightforward. In particular, we predict such a chain to adopt extended conformations beyond what it would be expected for a Gaussian chain^86^ (Figure 6B), also reflected by the markedly long persistence length of the order of 10 nm (20 monomers) (Figure 6D). This is likely due to strong self-repelling electrostatic forces, promoting solvent–glycan interactions over glycan–glycan interactions (Figure 6C). It should be noted that such an estimate could not have been obtained from the simulations of short fragments of size comparable to the persistence length.

When solvated by explicit Martini water beads and ions, the 123mer CSA chain system consisted of almost 1 million CG beads. One microsecond molecular dynamics analysis of such a system on a 2 GPU and 72 CPU node required 7 days of computing. If treated atomistically, such simulation would have implied roughly 11 million atoms. Although Martini CG and all-atom time-scales are not directly comparable,^90^ microsecond-long simulations of such a system would be extremely time- and resource-consuming, requiring approximately 500 days of computing using a single 128-CPU node.^98^ We thus provide evidence of how our model enables access to spatial and temporal scales that are currently extremely difficult with atomistic simulations.

Despite accurately capturing single-chain properties, simulations of multiple chains, using the recommended Martini 3 interaction and simulation parameters, resulted in aggregation (Figure 4). We attribute this tendency to the strong electrostatic interaction between the negatively charged sugar groups and sodium ions, which promotes interchain association of the CSA chains bridged by sodium ions (Figure S6). Similar ion-induced polymeric assembly has been observed in implicit-solvent simulations of disordered mucin glycoproteins.^99^ To address this issue, we systematically varied three key electrostatic properties of the system that attenuated the strong interactions with sodium and quantified their impact on the aggregation of CSA (Figure 4B). By using the reaction-field scheme, we demonstrated that aggregation could be prevented by introducing two changes simultaneously: increasing the size of ion beads, reflecting their hydration shell (as it was originally implemented in previous Martini version^90^), and rescaling charge of salt ions, according to previously proposed continuum correction to take into account electrostatic screening of water.^58^ Charge scaling is actually preferable to directly changing the screening constant, as it does not affect all other Coulombic interactions of the system. It should be noted that charge scaling and calibrated Lennard-Jones parameters of ions have also been recently reported to properly describe ionic radial distribution functions in implicit-solvent CG simulations.^99^ By using PME, which is a more robust way to consider long-range electrostatic interactions, the extent of aggregation was overall reduced but not fully abolished, requiring a larger (hydrated) ion bead too. Furthermore, if the three conditions were in place, i.e., PME and bigger salt ion beads with rescaled charges, the CG model did not show aggregation, similarly to the atomistic reference. Accordingly, we provide at least three schemes of varying level of modifications (in reference to default Martini 3 force field) to attenuate glycan–cation interactions and thereby prevent CSA aggregation (Figure 4C). It should be noted that aggregation has been reported in previous simulations with Martini (version 2.2).^47,100,101^ Rather than rescaling Lennard-Jones parameters, as previously proposed for polysaccharides,^47,101^ we here propose tuning electrostatic interactions as an approach to mitigate it.

Treatment of electrostatics of highly charged systems is a challenging task both in all-atom^58^ and coarse-grained MD simulations. Because CSA is a highly sulfated and thus highly negatively charged biopolymer, studying its aggregation becomes an excellent test to assess electrostatics in MD simulations. Already at the all-atom level, electrostatics that govern CSA interaction with ions have been found difficult to reproduce and to be highly sensitive to force-field parameters.^33^ At the coarse-grained level, ions have been shown to impact the polymeric assembly of a mucin disordered glycoprotein in implicit-solvent CG simulations.^99^ We here demonstrate the impact that long-range electrostatics (reaction-field vs PME), ion solvation (small vs big CG ion beads), and electric screening (fractional ionic charges) have on clustering of CSA. From this we recommend that, whenever possible, Martini 3 simulations of CSA should use PME, with ±0.75 Q5 beads for monovalent salt ions. This setup, however, has two drawbacks: PME is more computationally demanding than the reaction-field. In addition, simulations of ion binding molecules may not be possible with implicitly hydrated ion beads. In such cases, we suggest considering the other two nonaggregating conditions as an alternative. Finally, our data also demonstrate that regular Martini 3 parameters are already adequate for simulations of short CSA single chains in highly diluted conditions, where aggregation does not pose issues. In addition, these changes may be considered for other Martini 3 systems, where artifactual aggregation is observed. Although the presented study focuses on CSA, we believe that it provides some practical clues for future studies, aiming at solving the issues existing with electrostatics in Martini, e.g., with cations, not only for the simulation of glycans but more generally for studying highly charged systems.

We based our model on the Martini 3 force field as it enables simulations of large and complex multicomponent systems in coarse-grained resolution. This allowed us to simulate multiple (Figure 4) and also large (Figure 6) CSA chains in explicit solvent. We thereby complement existing Martini models of Glycosaminoglycans,^47^ available for the 2.2 version, by providing here a model of CSA, the most common CS form, compatible with the Martini 3 version. Considering the solvent explicitly may constitute an advantage over existing implicit-solvent models of CSA,^43–46^ when evaluating solvent properties such as viscosity or friction. This will be of relevance to study processes such as lubrication, load bearing of joints, or shear-mediated cellular adherence. In addition, our model can be used in conjunction with models of other biomolecules, such as proteins. We demonstrate this by simulating the VAR2CSA–CSA complex (Figure 5). This constitutes another advantage of our model, as biologically relevant questions related to interactions of CSA with other biomolecules can now be addressed within the Martini 3 framework. In the particular case of VAR2CSA–CSA, it will be highly interesting to assess how electrostatics, e.g., via changes in the ionic strength, could impact the balance between protein–glycan and protein–protein interactions, potentially influencing the force-mediated multivalent binding kinetics of this complex.^40,94^

In conclusion, we have developed here a Martini 3 coarse-grained model of chondroitin sulfate A that allows its MD simulations at reduced computational cost without compromising simulation accuracy. This opens the door for exciting research to study CSA and potentially other chondroitin sulfates.

## Supplementary Material





## Notes

Distance comparison; comparison of bond length distributions; comparison of angle distributions; comparison of dihedral angle distributions; performance of CG CSA simulation; comparison of pairwise distance distributions; comparison of radial distribution functions; *C*_α_ root mean square deviation; time traces of end-to-end distance fluctuations; decay of autocorrelation *C*(*x*) functions; analysis of glycosidic bond conformers; comparison of polymer size descriptors; and convergence of measurements (PDF)
Trajectory of CSA 123mer under equilibrium conditions (MP4)
