# Supplementary material for: Coarse-Grained Martini 3 Model of Chondroitin Sulfate A
Source: J Chem Theory Comput. 2026 Feb 23;22(5):2622–34. doi: 10.1021/acs.jctc.5c01743 (PMC12980720; doi:10.1021/acs.jctc.5c01743)
Supplement: Supplementary file 1 [file ct5c01743_si_001.pdf]

Supporting information: Coarse-grained Martini 3 model of chondroitin sulfate A

Paulius Greicius,[†,‡,¶] Frauke Gräter,[†,‡,¶] Fabian Grünewald,[¶] and Camilo Aponte-Santamaría[*,†,¶]

†*Max Planck Institute for Polymer Research, Ackermannweg 10, Mainz, Germany*

‡*Max Planck School Matter to Life, 69120 Heidelberg, Germany*

¶*Heidelberg Institute for Theoretical Studies (HITS), Schloss-Wolfsbrunnenweg 35, Heidelberg, Germany*

E-mail: apontec@mpip-mainz.mpg.de

Comparison of sugar ring bead bond distance distributions between forward-mapped all-atom and Martini 3 trajectories (Figure S1), comparison of all other bond distances (Figure S2), angles (Figure S3) and dihedrals (Figure S4). Evaluation of coarse-grained Martini 3 model accuracy in simulations with default Martini 3 parameters (Figure S5). Comparison of distance distributions between a pair of residues in the CSA chain (Figure S6). Comparison of sodium cation densities in all-atom and coarse-grained trajectories (Figure S7). $C\alpha$ root mean square deviation time traces of VAR2CSA coarse-grained simulations (Figure S8). Time traces of end-to-end distance of CSA 123mer in coarse-grained equilibrium simulations (Figure S9). Autocorrelation function of GAL or GLA residues form 123mer equilibrium simulations (Figure S10). Glycosidic bond angles from all-atom equilibrium simulations of CSA 21mer and the equivalent forward-mapped dihedral angle (Figure S11). Comparison of polymer size descriptors under difference simulation setups (Figure S12). Comparison of cation radial distribution functions around anionic sugar groups under different simulation

setups (Figure S13). Convergence of measurements in all-atom simulations (Figure S14). Example equilibrium dynamics trajectory of CSA 123mer (Supporting Movie 1).

Supporting Figures

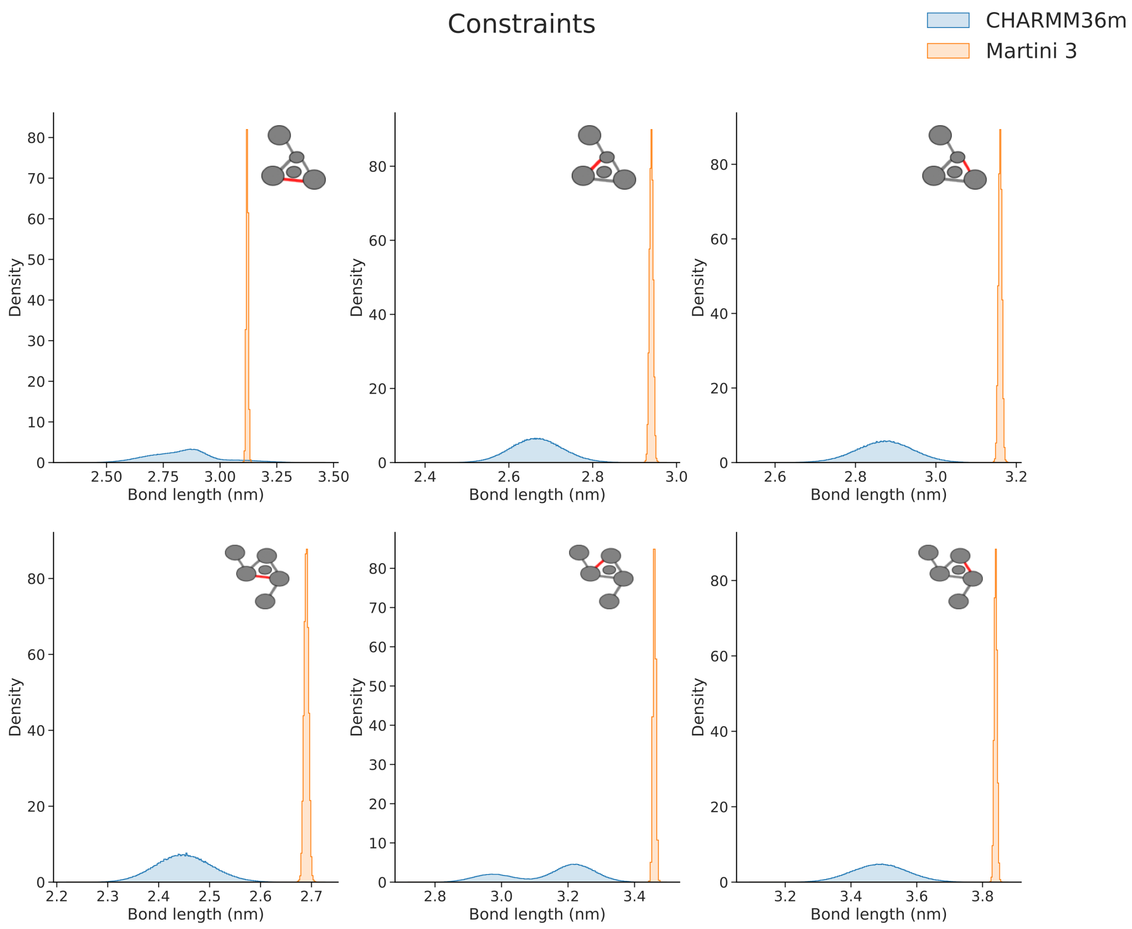

Supporting Figure 1. Ring bonds are represented as distance constraints in Martini 3 model. Distance comparison between forward mapped all-atom trajectory (CHARMM36m) and Martini 3. Inlet shows the corresponding bond marked in red.

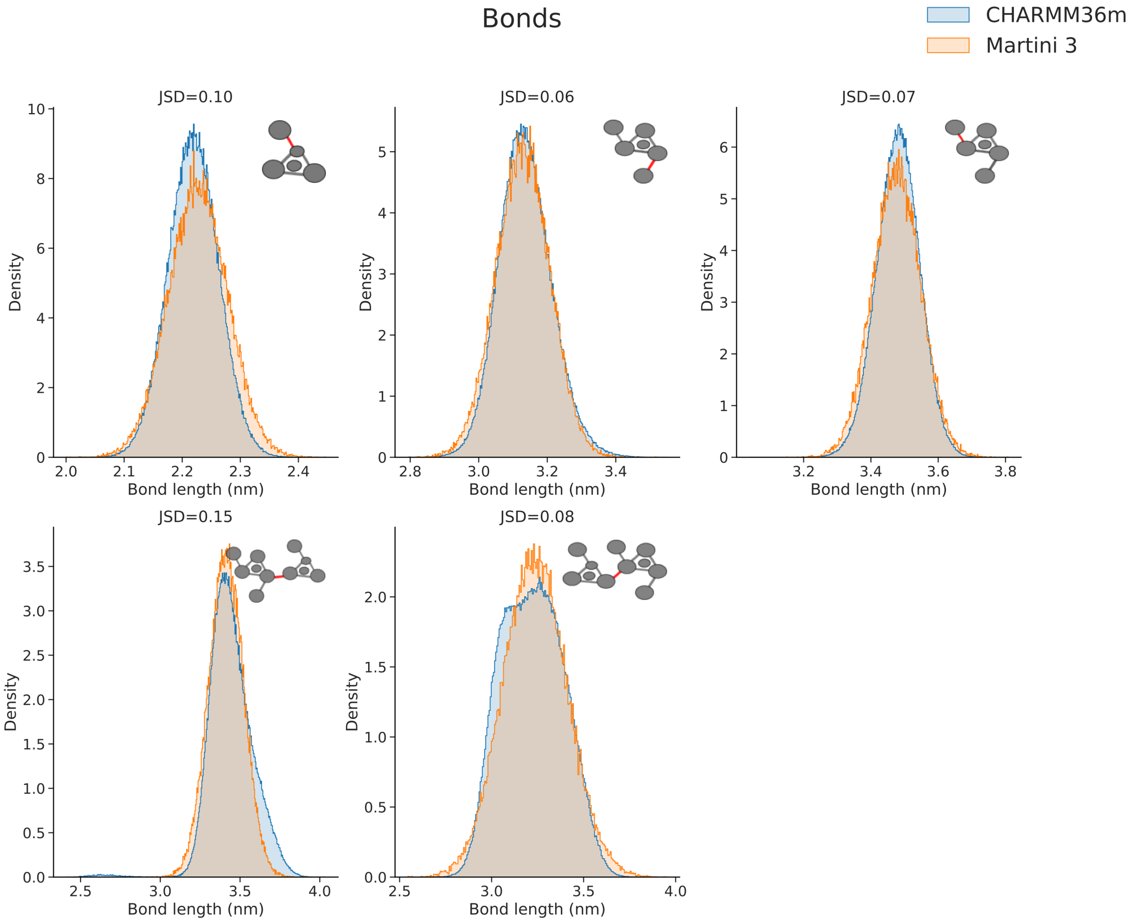

Supporting Figure 2. Comparison of bond length distributions. Bond length comparison between forward mapped all-atom trajectory (CHARMM36m) and Martini 3. Inlet shows the corresponding bond marked in red.

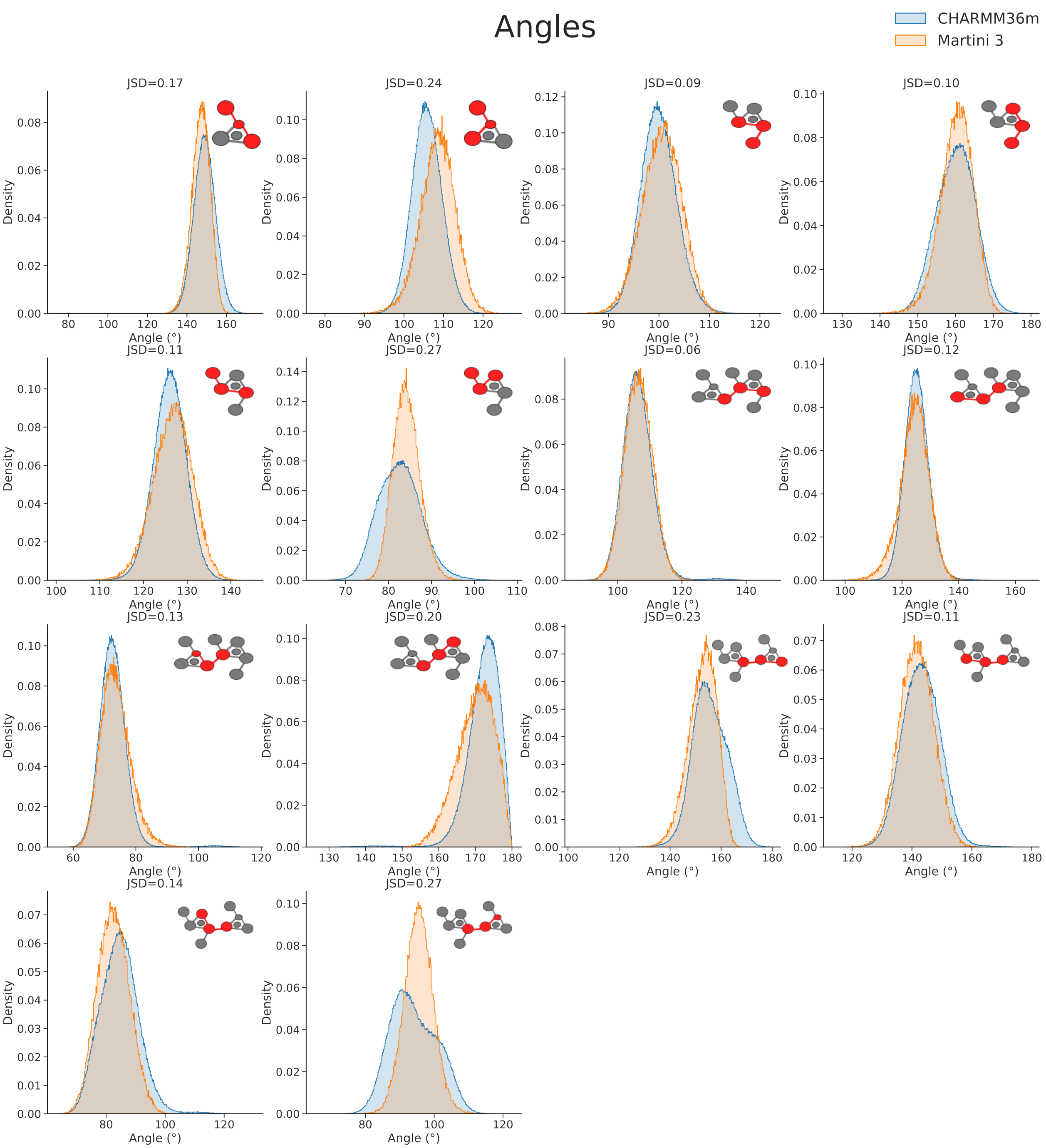

Supporting Figure 3. Comparison of angle distributions. Angle comparison between forward mapped all-atom trajectory (CHARMM36m) and Martini 3. Inlet shows the corresponding angle marked in red.

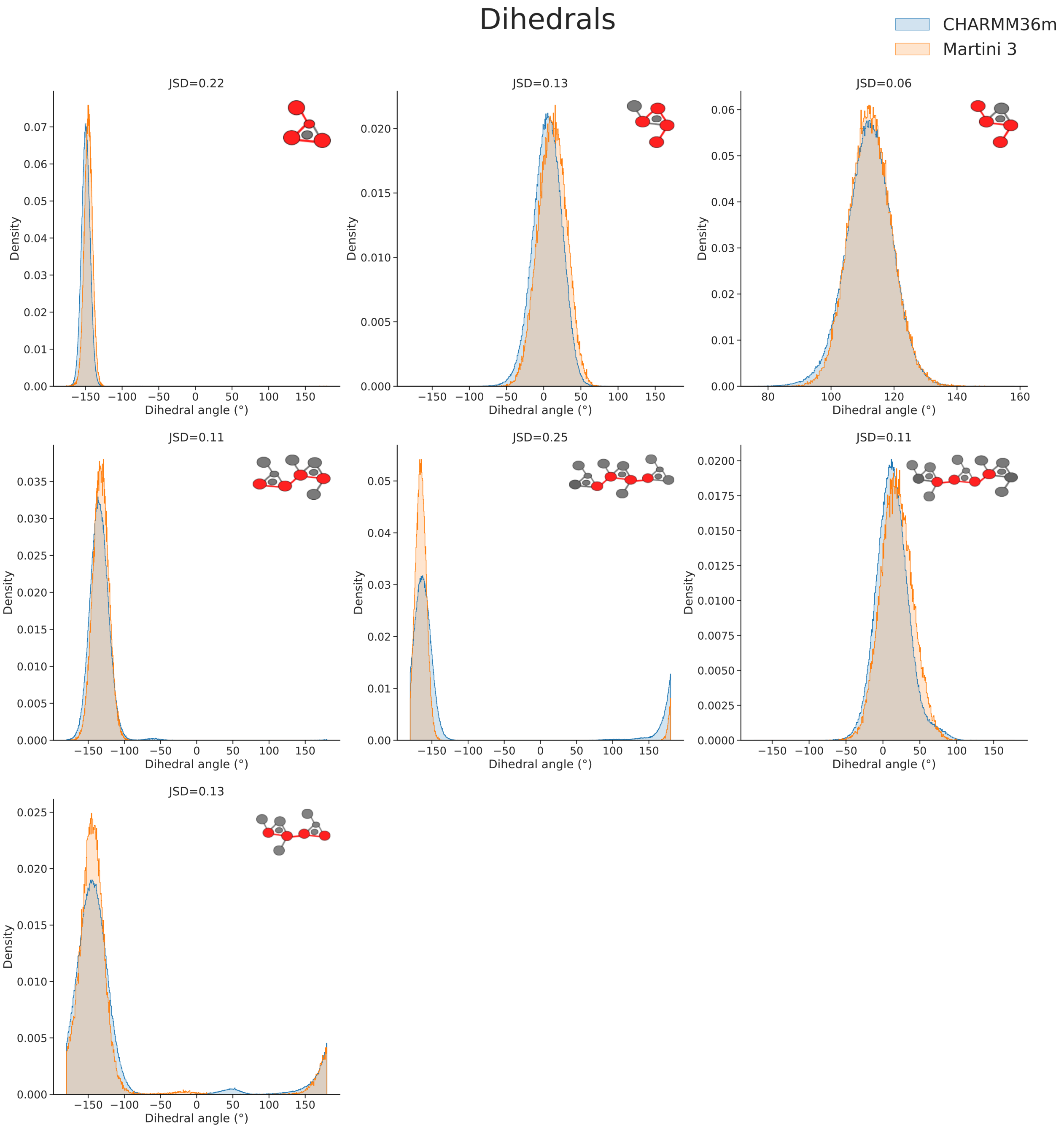

Supporting Figure 4. Comparison of dihedral angle distributions. Dihedral angle comparison between forward mapped all-atom trajectory (CHARMM36m) and Martini 3. Inlet shows the corresponding dihedral angle marked in red.

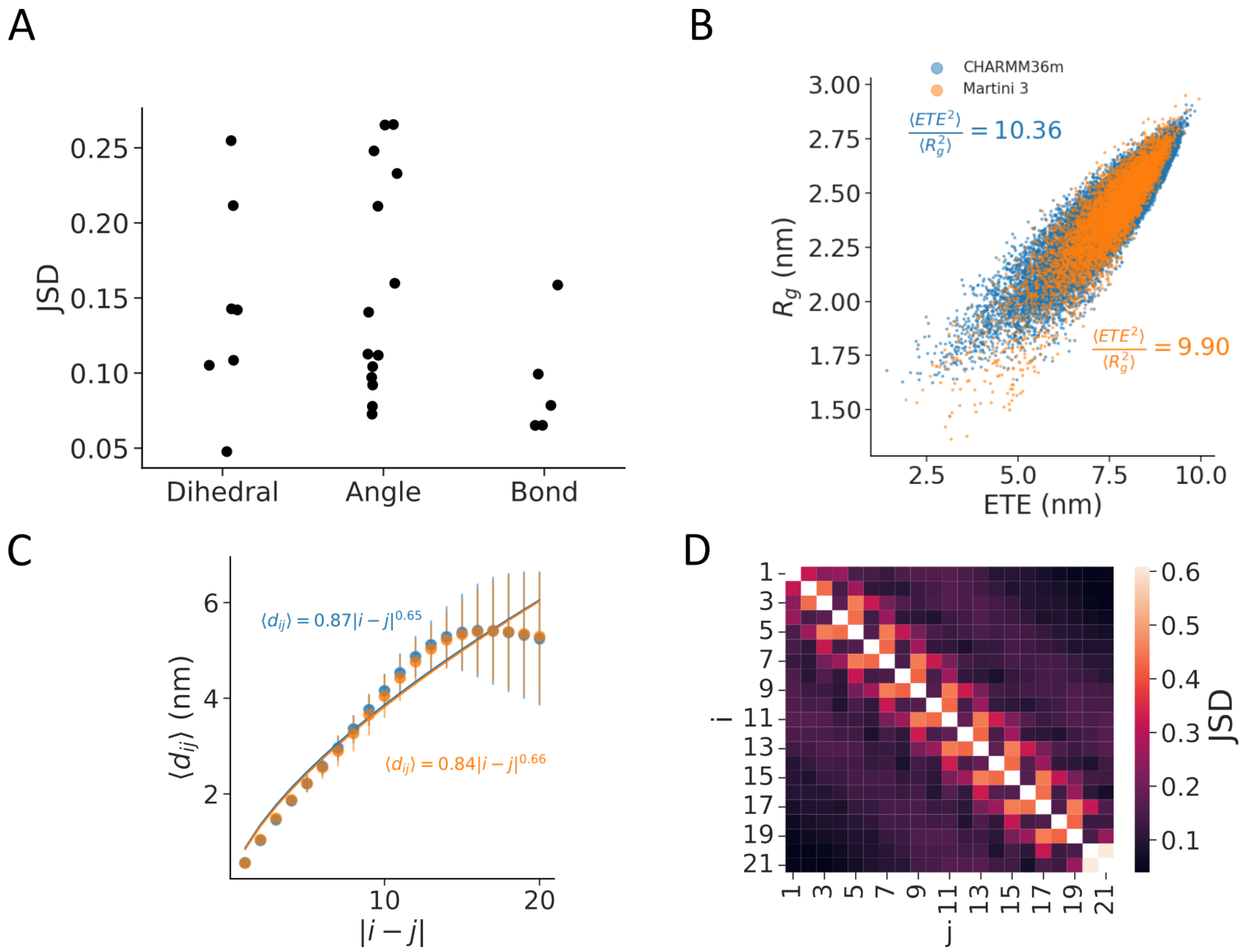

Supporting Figure 5. Performance of CG CSA simulation with default Martini3 parameters. (A) Jensen-Shannon divergence statistic for the three types of bonded terms used, comparing the Martini 3 model with the all-atom CHARMM36m reference data (JSD=0: identical distributions, and JSD large: dissimilar distributions). (B) Ratio between radius of gyration R_g and end-to-end distance ETE, recovered from MD simulations using all-atom (CHARMM36m) and Martini 3 models (color). The average squared ratio $\langle ETE^2 \rangle / \langle R_g^2 \rangle$ is indicated in both cases. (C) Average interresidue distance $\langle d_{ij} \rangle$ between the i-th and the j-th monomer of the chain (scatterplot: av.±s.e.). The line shows the fit $\langle d_{ij} \rangle = b|i-j|^{\nu}$, where b is proportionality constant in nm and ν is the scaling exponent. Resulting fitting parameters are indicated for both studied cases. Colors follow the same scheme as in B (D) JS divergence of interresidue distance distributions between all-atom and coarse-grained models.

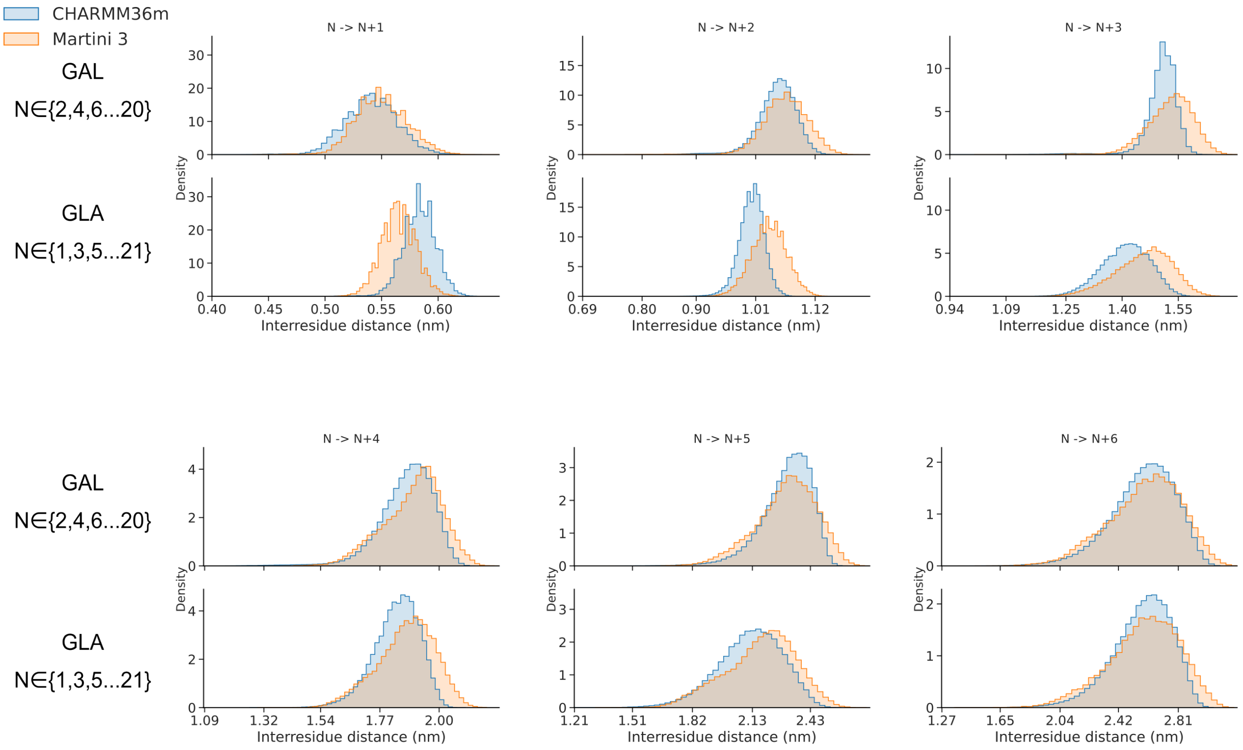

Supporting Figure 6. Comparison of pairwise distance distributions. Distance between CSA residue at position N and residues downstream of N in all-atom (CHARMM36m) and coarse-grained (Martini 3) simulations. Labels next to each row of plots indicate which sugar (GAL or GLA) was at position N. Title above the plot indicates which positions are compared.

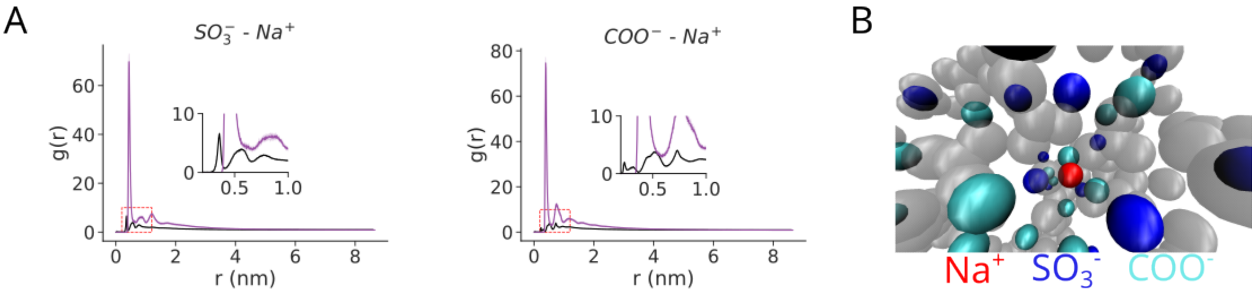

Supporting Figure 7. Sodium cation interactions are overestimated in coarse-grained simulations. (A) Comparison of radial distribution functions of sodium ions around carboxylic acid and sulfate groups. Inlet shows a zoom on the region marked by the red rectangle. Black line marks RDFs from CHARMM36m simulations, purple line - Martini 3 simulations with default parameters (PME and ±1.0 TQ5 salt beads). (B) Snapshot of sodium cation (red) sandwiched inside CSA aggregate (gray), between negatively charged sulfate (blue) and carboxylic acid (cyan) beads.

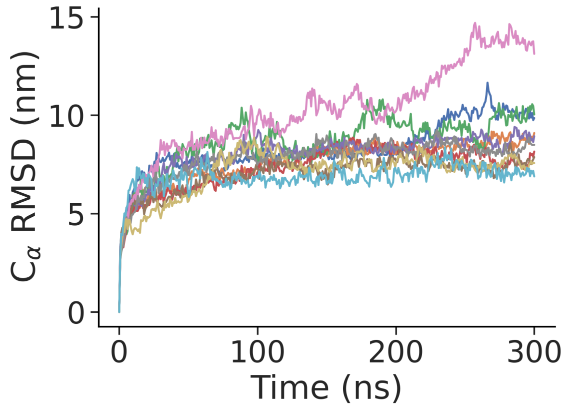

Supporting Figure 8. C_α **root mean square deviation (RMSD) of VAR2CSA coarse-grained model** Different colors show n=10 independent replicates.

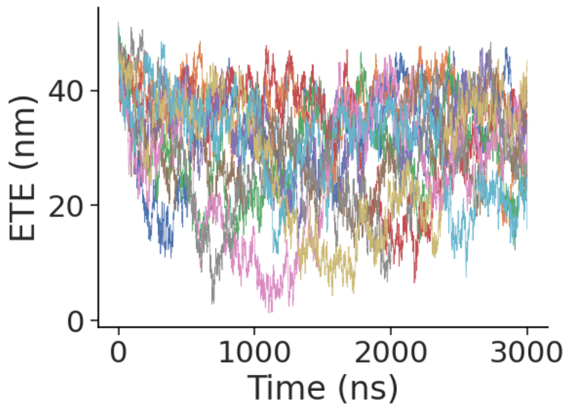

Supporting Figure 9. Time traces of end-to-end distance fluctuations of CSA 123mer. Different colors represent individual simulation replicates.

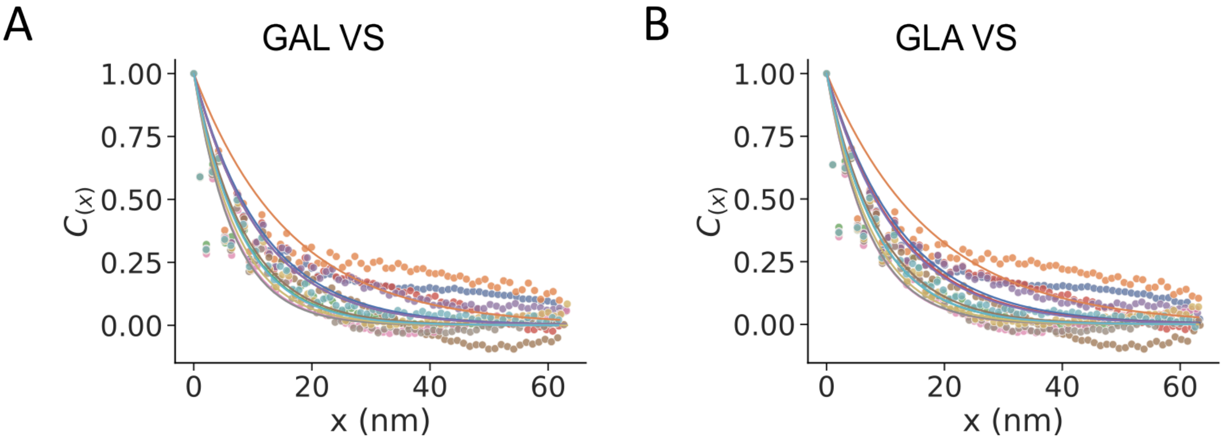

Supporting Figure 10. Decay of autocorrelation C(x) functions in 123mer CSA chain Comparison of autocorrelation functions of GAL virtual sites (A) and GLA virtual sites (B) with distance x (simulation data: points and exponential decay fit: lines). Distance at which C(x)=0.5 was considered the persistence length, in A this was $9.6 \pm 2.8\,$nm, in B - $10.3 \pm 3.2\,$nm (average $\pm$ s.d.). Different colors represent $n = 10$ independent replicates.

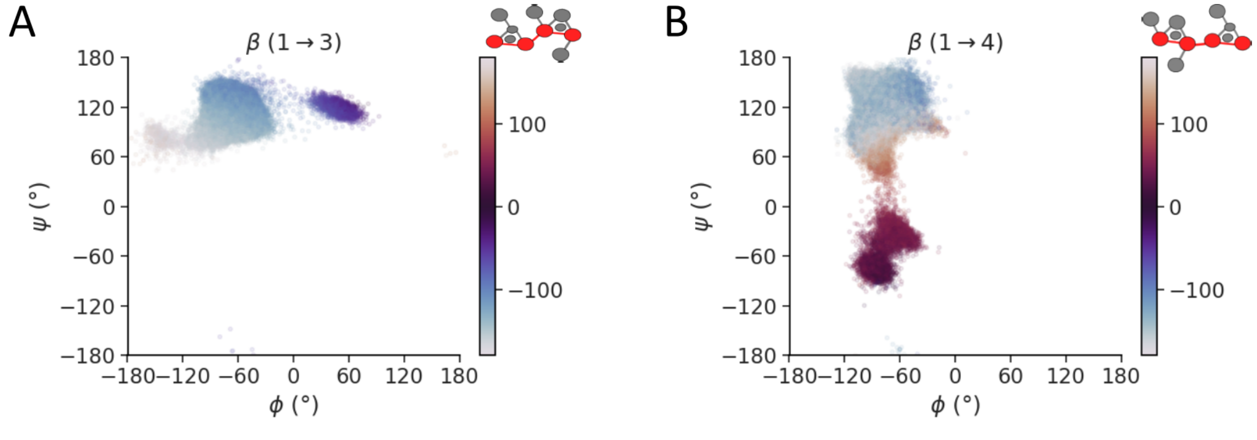

Supporting Figure 11. Analysis of glycosidic bond conformers. Values for ϕ and ψ angles of glycosidic bond β1,3 between GAL and GLA (A) and β1,4 between GLA and GAL in all-atom (CHARMM36m) simulations. Scatter points are colored by the corresponding dihedral angle from forward mapped trajectory. Inlet next to the colorbar marks the dihedral.

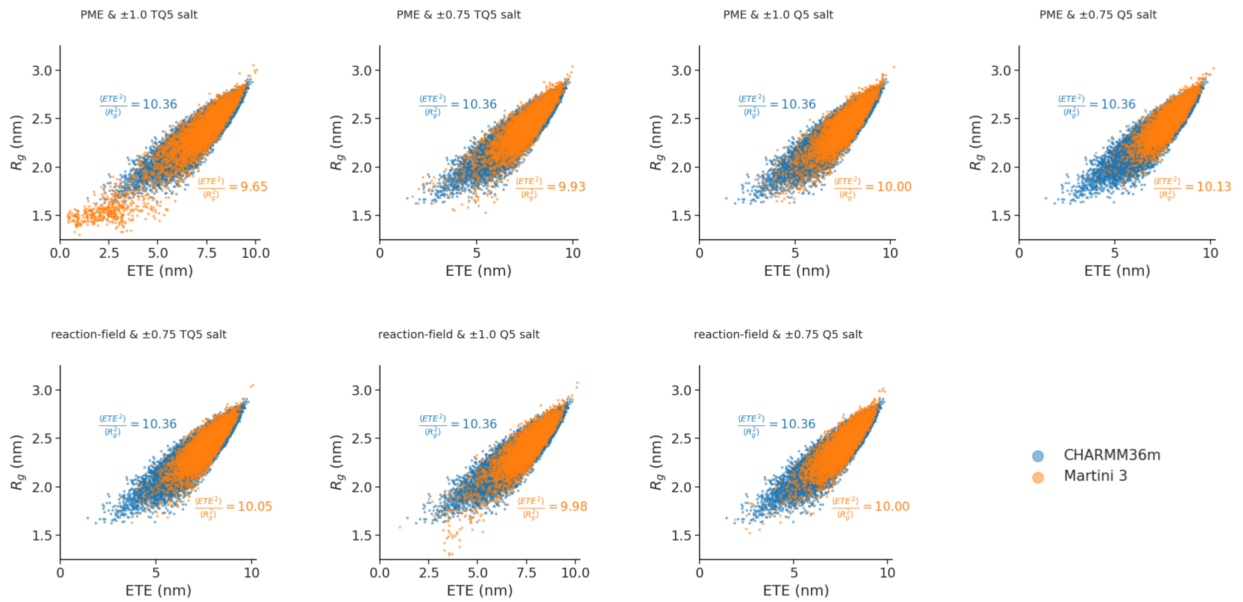

Supporting Figure 12. Comparison of polymer size descriptors under different simulation setups. Ratio between radius of gyration R_g and end-to-end distance ETE, recovered from MD simulations of all-atom (CHARMM36m) or coarse-grained (Martini 3) CSA 21mer models. Panels show data of Martini 3 simulations using different parameter combinations: PME or reaction-field, salt ion charge of ±1.0 or ±0.75 and choice for salt ion beads - TQ5 or Q5, as indicated in the figure titles. For comparison, all-atom data generated with recommended CHARMM36m parameters is shown (same data in each panel). The average squared ratio $\langle ETE^2 \rangle / \langle R_g^2 \rangle$ is indicated in for both models (color).

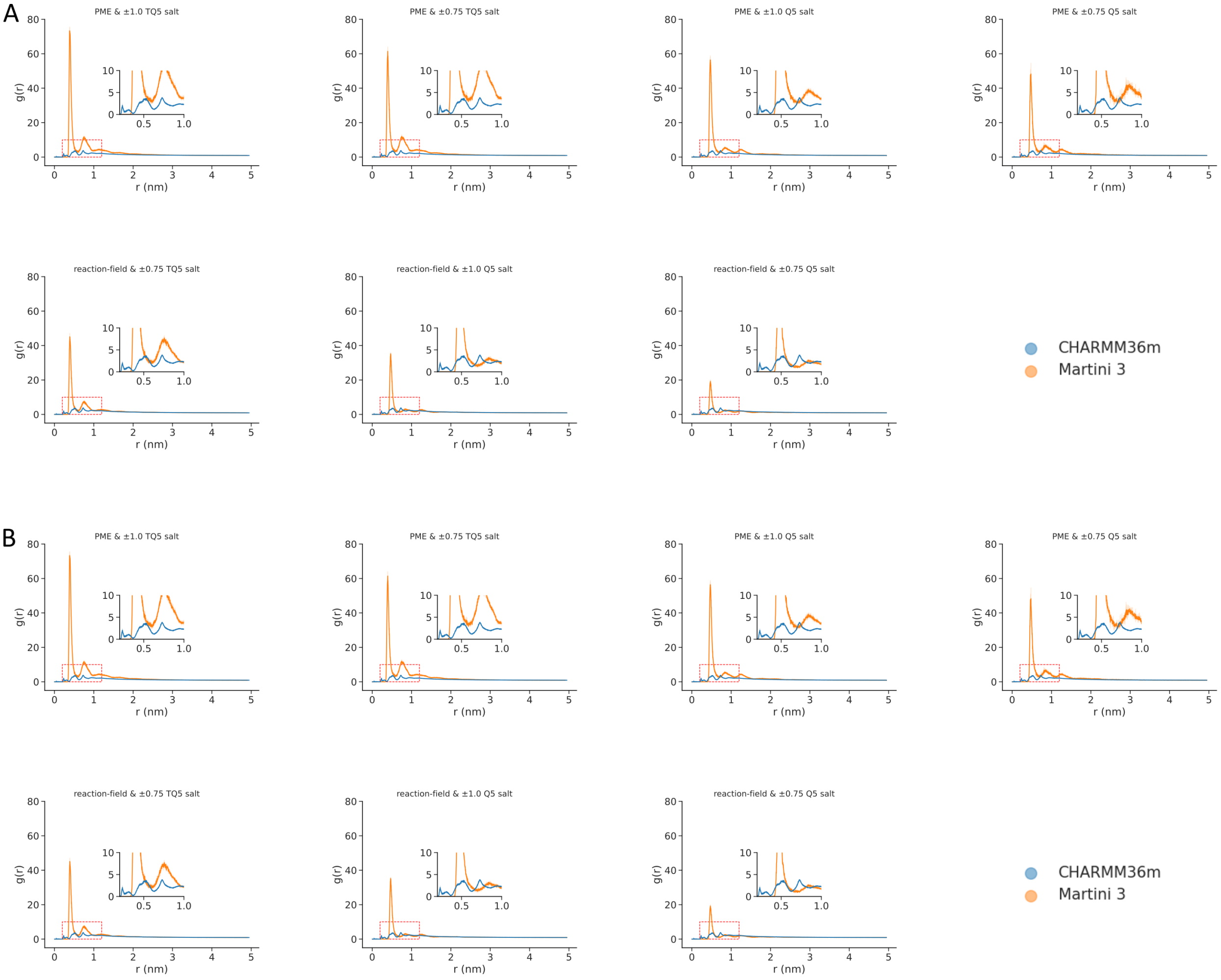

Supporting Figure 13. Comparison of radial distribution functions of cations around anionic groups under different simulation setups Figure shows RDFs of sodium ions around carboxylic acid (A) and sulfate (B) groups from MD simulations of all-atom (CHARMM36m) or coarse-grained (Martini 3) CSA 21mer models. Panels show data of Martini 3 simulations using different parameter combinations: PME or reaction-field, salt ion charge of ±1.0 or ±0.75 and choice for salt ion beads - TQ5 or Q5, as indicated in the figure titles. For comparison, all-atom data generated with recommended CHARMM36m parameters is shown (same data in each panel). Inlet shows a zoom on the region marked by the red rectangle.

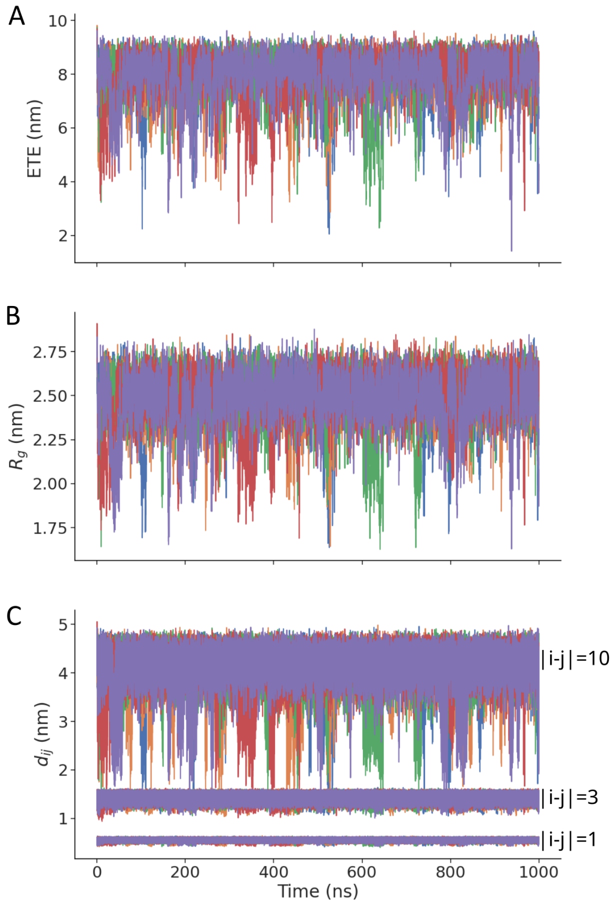

Supporting Figure 14. Convergence of measurements in all-atom simulations Figure shows time-traces for end-to-end distance (A), radius of gyration (B) and representative inter-saccharide distances (C) from all-atom (CHARMM36m) simulations. Colors mark different replica. Text next to traces in C indicate which inter-saccharide pair is shown.

Supporting Movie 1. Trajectory of CSA 123mer under equilibrium conditions. D-glucuronic acid (GLA) is colored in pink and the N-acetyl-D-galactosamine 4-sulfate (GAL) in blue. Salt ions and water molecules not shown.
